# Dephospho-Coenzyme A Kinase Is an Exploitable Drug Target against Plasmodium falciparum: Identification of Selective Inhibitors by High-Throughput Screening of a Large Chemical Compound Library

**DOI:** 10.1128/aac.00420-22

**Published:** 2022-10-31

**Authors:** Arif Nurkanto, Riyo Imamura, Yulia Rahmawati, Erwahyuni Endang Prabandari, Danang Waluyo, Takeshi Annoura, Kazuki Yamamoto, Masakazu Sekijima, Yuki Nishimura, Takayoshi Okabe, Tomoo Shiba, Norio Shibata, Hirotatsu Kojima, James Duffy, Tomoyoshi Nozaki

**Affiliations:** a Research Center for Biosystematics and Evolution, Research Organization for Life Sciences and Environmental, National Research and Innovation Agency (BRIN), Cibinong, Indonesia; b Graduate School of Medicine, The University of Tokyo, Tokyo, Japan; c Drug Discovery Initiative (DDI), The University of Tokyo, Tokyo, Japan; d Research Center for Vaccine and Drug, Research Organization for Health, National Research and Innovation Agency (BRIN), Cibinong, Indonesia; e Department of Parasitology, National Institute of Infectious Diseases (NIID), Tokyo, Japan; f Department of Computer Science, Tokyo Institute of Technology, Tokyo, Japan; g Department of Biological Sciences, Graduate of School of Sciences, The University of Tokyo, Chiba, Japan; h Department of Applied Biology, Graduate School of Science and Technology, Kyoto Institute of Technology, Kyoto, Japan; i Department of Nanopharmaceutical Sciences, Nagoya Institute of Technology, Nagoya, Japan; j Medicines for Malaria Venture, International Center Cointrin, Geneva, Switzerland

**Keywords:** malaria, coenzyme A, high-throughput screening, dephospho-CoA kinase, *Plasmodium falciparum*, inhibitor, antimalarial agents

## Abstract

Malaria is a mosquito-borne fatal infectious disease that affects humans and is caused by *Plasmodium* parasites, primarily Plasmodium falciparum. Widespread drug resistance compels us to discover novel compounds and alternative drug discovery targets. The coenzyme A (CoA) biosynthesis pathway is essential for the malaria parasite P. falciparum. The last enzyme in CoA biosynthesis, dephospho-CoA kinase (DPCK), is essential to the major life cycle development stages but has not yet been exploited as a drug target in antimalarial drug discovery. We performed a high-throughput screen of a 210,000-compound library using recombinant P. falciparum DPCK (*Pf*DPCK). A high-throughput enzymatic assay using a 1,536-well platform was developed to identify potential *Pf*DPCK inhibitors. *Pf*DPCK inhibitors also inhibited parasite growth in a P. falciparum whole-cell asexual blood-stage assay in both drug-sensitive and drug-resistant strains. Hit compounds were selected based on their potency in cell-free (*Pf*DPCK) and whole-cell (*Pf*3D7 and *Pf*Dd2) assays, selectivity over the human orthologue (*Hs*COASY) and no cytotoxicity (HepG2). The compounds were ranked using a multiparameter optimization (MPO) scoring model, and the specific binding and the mechanism of inhibition were investigated for the most promising compounds.

## INTRODUCTION

Malaria is a protozoan infection caused by *Plasmodium* species and transmitted by mosquito bites. Malaria causes approximately an estimated 241 million cases and kills an estimated 627,000 people in 2020, mainly in tropical and subtropical regions worldwide, representing more cases and deaths than in 2019 (1). Plasmodium falciparum is the most dangerous and deadliest among five human-infecting species. Despite significant global health investments in recent years, which decreased malaria mortality by more than 50% between 2000 and 2016, malaria remains one of the major infectious killers. This is largely attributable to the lack of an effective vaccine and the emergence of resistance to virtually all available antimalarial chemotherapeutics, including the standard chemotherapeutic regimens, which include artemisinin and its partner drugs in artemisinin combination therapies (ACT). Thus, new antimalarial agents that act on different targets, preferably in multiple life cycle stages, and have novel mechanisms of action, are urgently needed to overcome the lack or shortage of effective therapeutic options (2–5).

**FIG 1:**
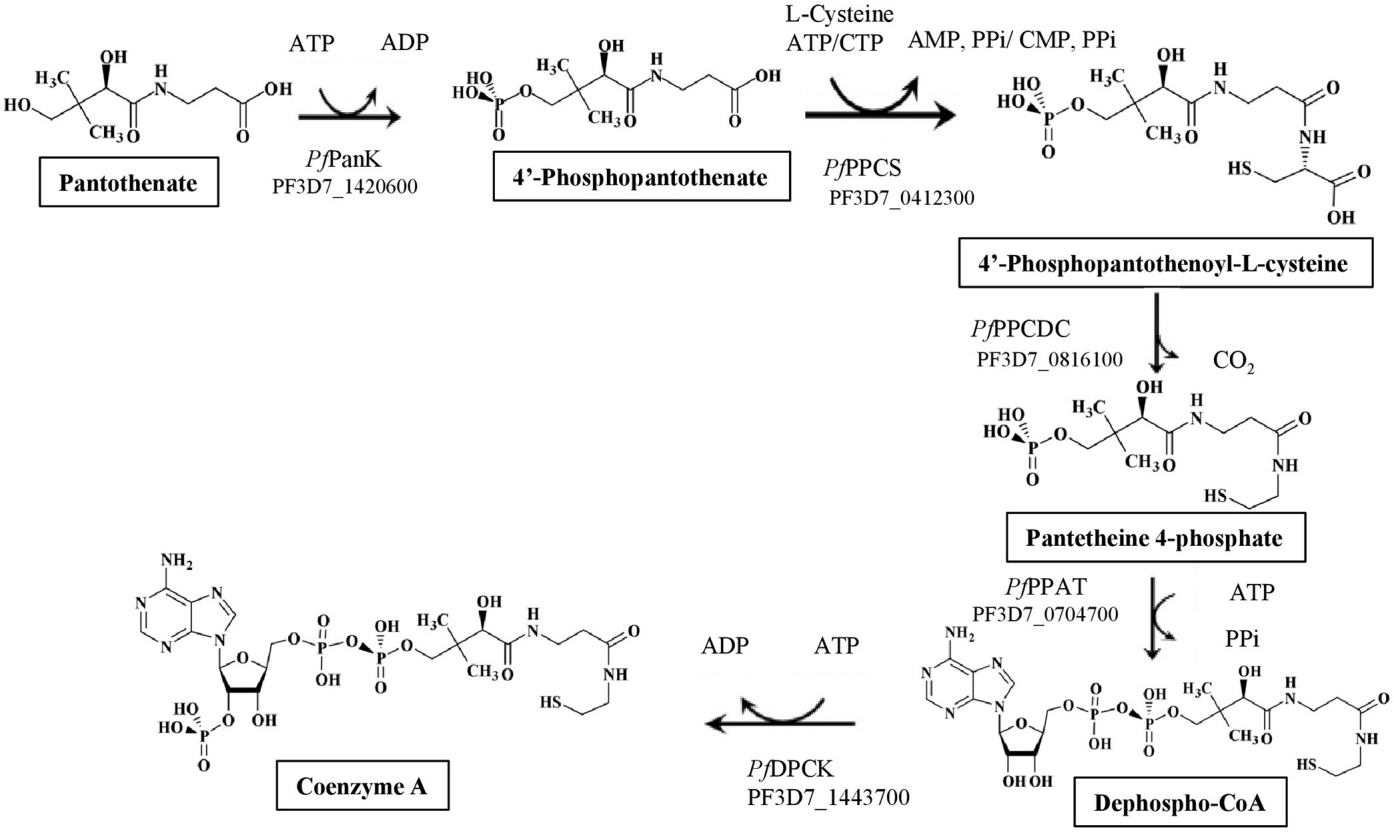
Coenzyme A biosynthetic pathway in P. falciparum. ID numbers of individual enzymes in PlasmoDB are also shown. *Pf*PanK, P. falciparum pantothenate kinase; *Pf*PPCS, P. falciparum phosphopantothenoylcysteine synthetase; *Pf*PPCDC P. falciparum phosphopantothenoylcysteine decarboxylase; *Pf*PPAT, P. falciparum phosphopantetheine adenylyltransferase; *Pf*DPCK, P. falciparum dephosphocoenzyme A kinase.

One of the validated and unexplored drug targets for the development of antimalarials is the coenzyme A (CoA) biosynthetic pathway (6). CoA is an essential cofactor that acts as an acyl group carrier and involved in approximately 9% of all (3,500) cellular activities (https://www.brenda-enzymes.info/). CoA is synthesized by 4 to 5 enzymatic steps (Fig. 1), and the pathway is initiated by a conversion of pantothenic acid (vitamin B_5_) to 4-phosphopantothenate, followed by later steps in which l-cysteine and pyrimidine/purine nucleotides are used as the substrates (7). Some bacteria, archaea, mammals, and plants are able to produce pantothenate *de novo* (8–10), while the others can scavenge it from the environment, including the hosts or prey (8, 11). The first enzyme in the pathway, pantothenate kinase (PanK), has been well characterized and considered to be a rational drug target against P. falciparum (8, 11, 12). The last enzyme in this pathway, dephospho-CoA kinase (DPCK; EC 2.7.1.24), has also been proven to be essential, and it has been demonstrated that DPCK is rate limiting and allosterically regulated in P. falciparum (13, 14). Similarly, DPCK has been proven to be indispensable in the enteric protozoan Entamoeba histolytica and thus underpinned as a potential drug target (15). However, no specific inhibitors against P. falciparum dephospho-CoA kinase (*Pf*DPCK), which can be further developed for antimalarial drug discovery, have been documented.

In this study, we achieved expression and purification of *Pf*DPCK using an Escherichia coli expression system. We enzymatically characterized *Pf*DPCK and developed a high-throughput screening protocol to screen approximately 210,000 structurally diversified compounds from the Drug Discovery Initiative (DDI) library to discover potential inhibitors. Among these *Pf*DPCK inhibitors, 99 compounds (80%) showed growth inhibition toward both drug-sensitive and -resistant P. falciparum strains. The modes of inhibition of the representative inhibitors were determined. The specific binding and inhibition of *Pf*DPCK, but not its human counterpart, by the representative inhibitor was confirmed by *in silico* modeling of the two enzymes and the inhibitor.

## RESULTS

### Identification of DPCK from P. falciparum.

Since CoA plays an indispensable role in P. falciparum (16) and DPCK catalyzes the final committed step in its biosynthesis, we were prompted to identify and characterize the enzyme from Plasmodium falciparum. We found a single 825-bp-long protein coding sequence (PF3D7_1443700) encoding 274-amino-acid *Pf*DPCK with the calculated molecular mass of 31.9 kDa, from the genome database of the P. falciparum 3D7 strain (https://plasmodb.org/plasmo/).

DPCK is highly conserved among *Plasmodium* species. *Pf*DPCK exhibits 88 to 93% amino acid identity to Plasmodium vivax, Plasmodium malariae, Plasmodium ovale, Plasmodium knowlesi, and Plasmodium yoelii. *Pf*DPCK also shows the highest identity to, other than orthologs from *Plasmodium*, bacterial DPCK from Haemophilus influenzae and Aquifex aeolicus (33 and 30%, respectively). In contrast, similarity of *Pf*DPCK to the human counterpart (bifunctional coenzyme A synthase [*Hs*COASY]) is limited (24%). Phylogenetic analysis of 43 DPCK protein sequences based on 190 aligned positions inferred by maximum-likelihood analysis (see Fig. S1 in the supplemental material) also suggests that *Plasmodium* DPCKs are highly diverged from *Hs*COASY.

### Determination of kinetic properties of *Pf*DPCK.

**FIG 2:**
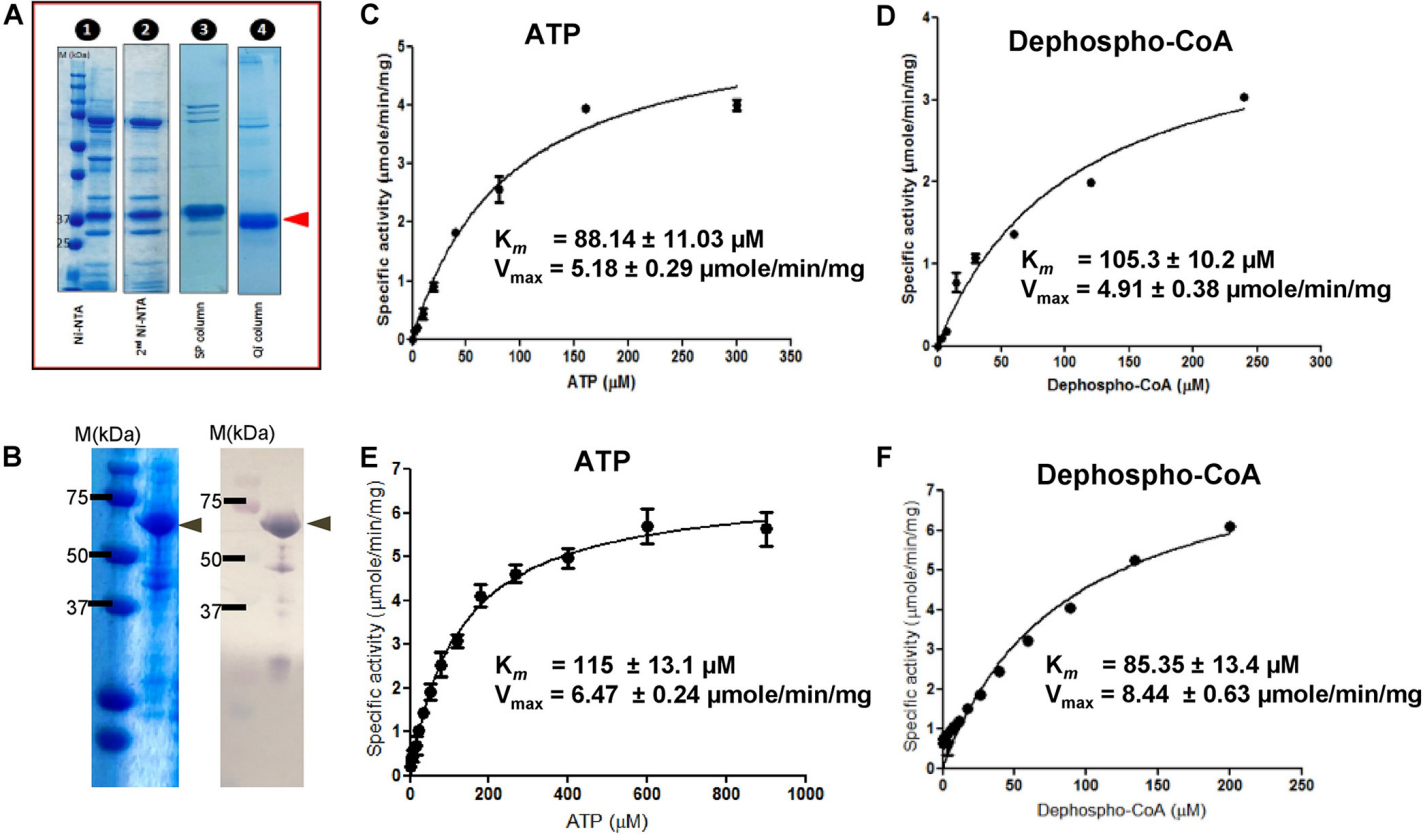
Biochemical characterization of recombinant *Pf*DPCK and *Hs*COASY. (A) Expression and purification of recombinant *Pf*DPCK. Samples at each step (lanes 1 to 4) of purification were subjected to 15% SDS-PAGE under reducing conditions and then stained with Coomassie brilliant blue R250. (B) Validation of the purity of recombinant *Hs*COASY. CBB staining (left) and immunoblot using anti-His antibody (right) are shown. (C to F) Kinetic analysis of *Pf*DPCK (C, D) and *Hs*COASY (E, F) to determine the *K_m_* values of ATP (C, E) and dephospho-CoA (D, F).

We successfully produced and purified recombinant *Pf*DPCK for enzymological characterization by using the E. coli expression system. The purity of the protein was estimated to be 90 to 95% by densitometric scanning of the Coomassie brilliant blue (CBB)-stained gels after SDS-PAGE (Fig. 2A). The apparent molecular mass of the recombinant *Pf*DPCK was consistent with the predicted mass of approximately 32 kDa plus 2.6 kDa corresponding to the histidine tag. The kinetic parameters of *Pf*DPCK were determined by measuring the initial rates obtained with different concentrations of ATP and dephospho-CoA (Fig. 2B and C). Both ATP and dephospho-CoA exhibited hyperbolic saturation kinetics. With the saturating concentrations of both substrates, *Pf*DPCK showed the apparent maximum rate of metabolism (*V*_max_) of 5.18 ± 0.29 μmol/min/mg. The *K_m_* values of *Pf*DPCK were 88.14 ± 11.03 μM and 105.3 ± 10.2 μM for ATP and dephospho-CoA, respectively. *Pf*DPCK was unable to utilize pantothenate, the precursor of CoA biosynthesis, as a substrate (the specific activity, <0.01 μmol/min/mg).

### Identification of potent *Pf*DPCK inhibitors by a high-throughput screening of the structurally diverse chemical library of 210,000 compounds.

**FIG 3:**
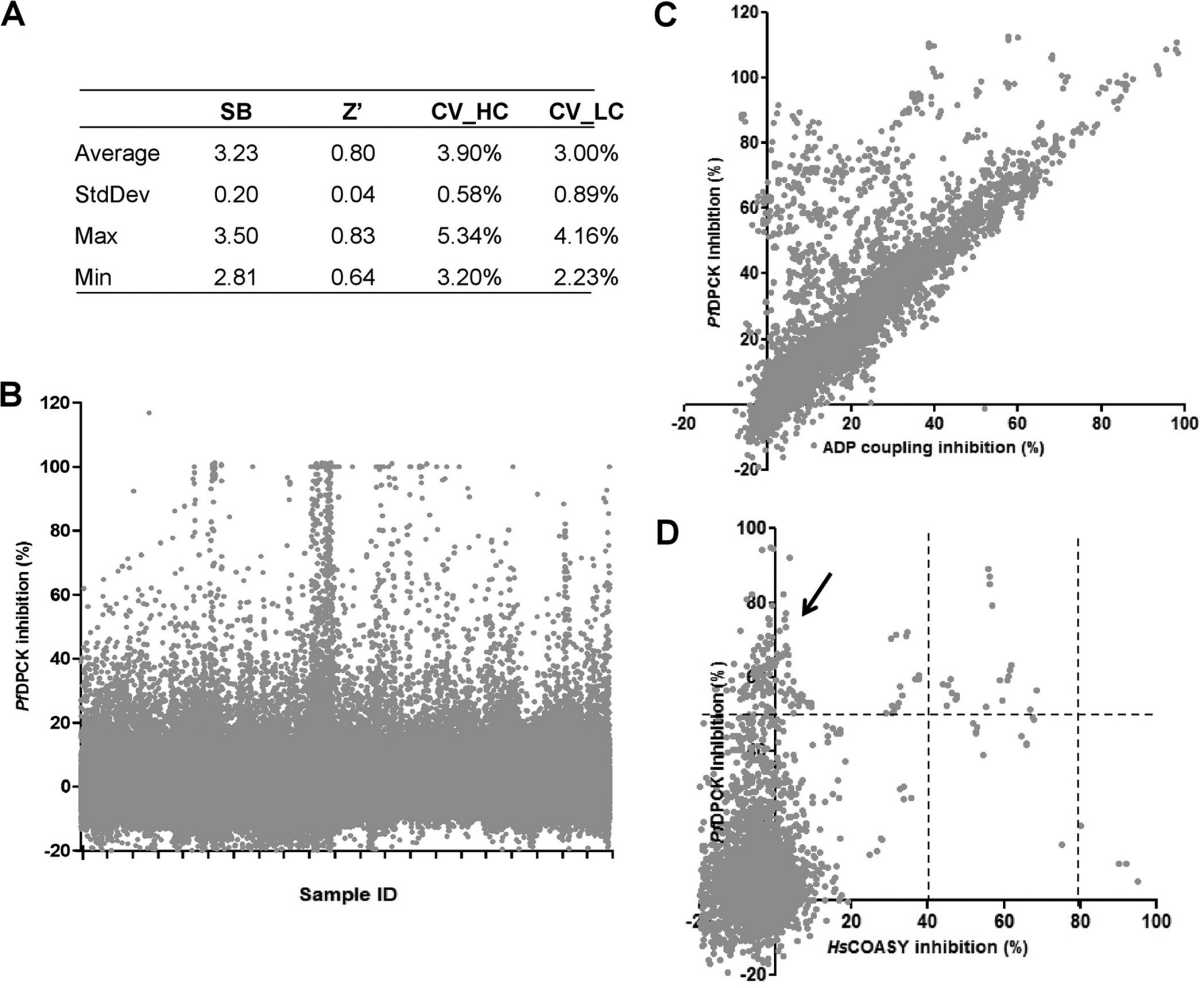
(A) Performance metrics of high-throughput screening of DDI library against *Pf*DPCK. Quality control values of all 150 1,536-well assay plates in the primary screening of the whole DDI library (210,000 compounds). The values of the S/B ratio (SB), Z′, coefficient of variation (CV) of positive control (CV_HC), and CV of negative control (CV_LC) are shown. (B) Plot showing percentage inhibition of *Pf*DPCK activity by all DDI compounds in the primary assay. (C) Scatterplot showing percentage inhibition of *Pf*DPCK activity (average of quadruplicate, *y* axis) and that of the ADP-coupled assay (*x* axis) of 825 secondary confirmed hits. (D) Scatterplot of percentage inhibition of *Pf*DPCK activity (the average of quadruplicate, *y* axis) and that of *Hs*COASY activity (*x* axis) of 359 tertiary hits. Arrowhead indicates compounds selected for titration assay.

**FIG 4:**
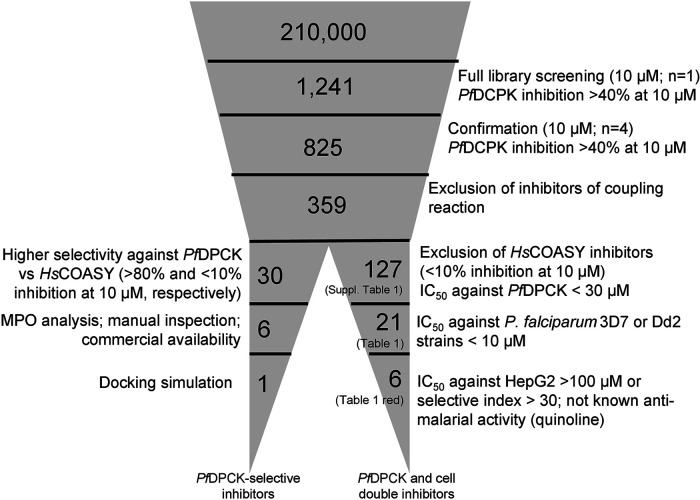
Screening cascade of the high-throughput screening of DDI library. The cascade is divided into two legs, screening based on the selectivity toward *Pf*DPCK over *Hs*COASY (left leg) and screening based on anti-erythrocytic-stage growth inhibition (right leg). The number of technical replicate assays is indicated (*n*). Individual steps of the cascade are described in the text.

In order to identify inhibitors against recombinant *Pf*DPCK, we successfully established the high-throughput screening protocol using a small reaction volume (2 μL) on a 1,536-well plate platform in a one-step enzyme-coupled fluorescence assay (17). A chemical library from the Drug Discovery Initiative, The University of Tokyo, Japan, composed of 210,000 structurally diverse compounds, was tested for *Pf*DPCK inhibition at a single final concentration of 10 μM. The screening was robust, with the mean Z′ factor (18) being 0.80 (Fig. 3A). With the selection criteria of >40% inhibition, 1,241 primary hits were identified with an overall hit rate of 0.6% (Fig. 3B; Fig. 4). In the second screening, the primary hits that showed >40% inhibition of *Pf*DPCK activity at 10 μM were tested in quadruplicate to exclude false positives that inhibited the coupled assay, but not *Pf*DPCK, which yielded 359 secondary hits (Fig. 3C). In the tertiary screening, the remaining hits were further tested against their human counterpart (*Hs*COASY) at a final concentration of 10 μM, and the hits that showed >10% inhibition toward *Hs*COASY were excluded to yield 127 tertiary hits, which were 0.06% from total compounds tested, which corresponds to 10.2% of the 1,241 primary hit compounds (Fig. 3D). The 50% inhibitory concentration (IC_50_) values against *Pf*DPCK and the 50% effective concentration (EC_50_) values against P. falciparum drug-sensitive 3D7, the chloroquine-resistant Dd2 strain, and human hepatoma HepG2 cells (19) of the 127 tertiary hits were determined using a five-dose titration assay in quadruplicate (Table S1).

We manually screened the 127 quaternary hits (Table S1) to further exclude undesirable hits, and we selected 21 compounds (Table 1). Our criteria of filtering were based on the previous experience of scientists at Medicines for Malaria Venture (MMV) and included the following: (i) being capable of inhibiting both *Pf*DPCK and P. falciparum erythrocytic-stage parasites of both drug-sensitive 3D7 and resistant Dd2 strains, (ii) not being frequent hits from other screening campaigns, (iii) relatively high selectivity against P. falciparum 3D7 and Dd2 strains compared to HepG2 human cell line (index > 30; either HepG2 to 3D7 or Dd2 or HepG2 to both strains), and (iv) the IC_50_ values being <10 μM.

**TABLE 1:** IC_50_ values of hit compounds against *Pf*DPCK enzyme, P. falciparum cell drug-sensitive (3D7) and drug-resistant (Dd2) strains, and liver human cell line (HepG2)^*a*^

Compound	Chemical structure	IC_50_ (μM)^*b*^ of:				Selectivity index	
		*Pf*DPCK	*Pf* cell 3D7	*Pf* cell Dd2	HepG2	IC_50_ (HepG2/3D7)	IC_50_ (HepG2/Dd2)
A-2	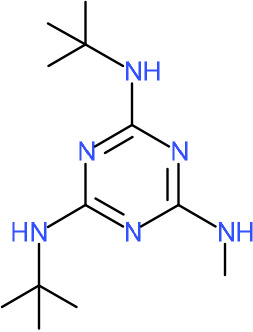	0.40 ± 0.07	4.43 ± 0.04	0.98 ± 0.50	>100	>23	>102
A-4	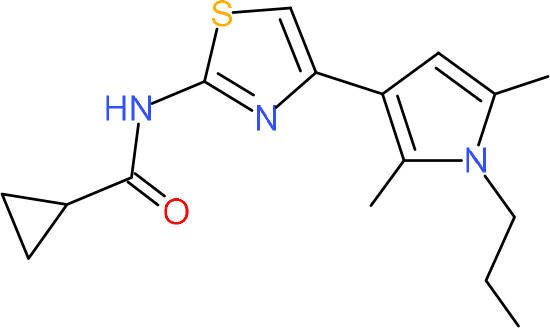	0.52 ± 0.09	4.45 ± 0.05	1.59 ± 0.23	ND	NA	NA
A-7	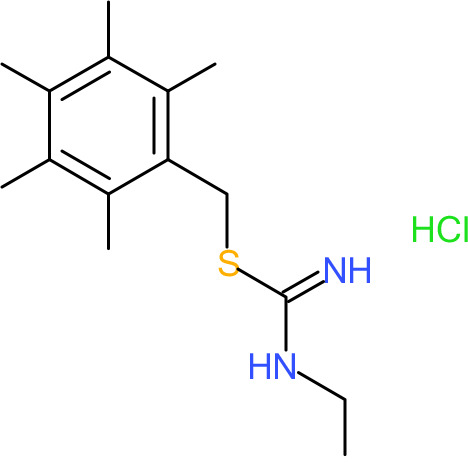	1.09 ± 0.03	1.10 ± 0.11	0.13 ± 0.01	9.04 ± 0.01	8.2	69.5
A-13	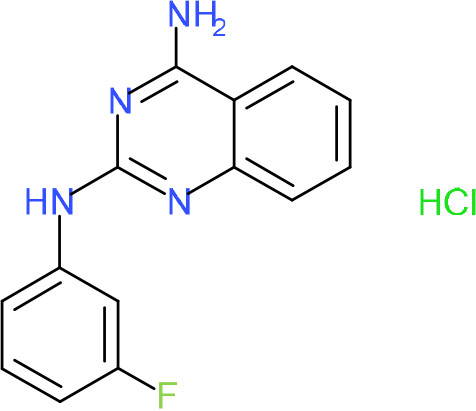	1.69 ± 0.05	1.30 ± 0.06	3.40 ± 0.35	ND	NA	NA
A-16	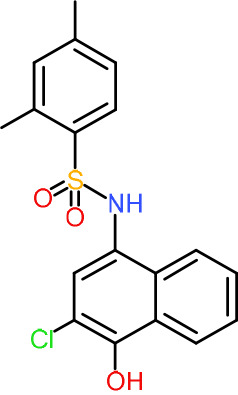	2.09 ± 0.04	2.21 ± 0.07	0.23 ± 0.01	39.64 ± 2.30	17.9	171.7
A-19	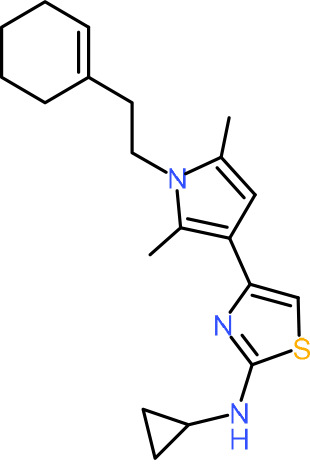	2.55 ± 0.03	3.64 ± 0.04	2.65 ± 0.13	81.50	22.4	30.8
A-22	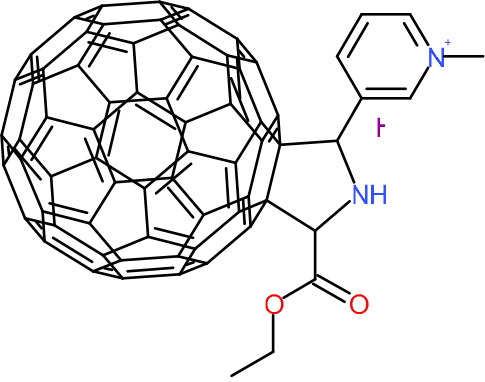	3.25 ± 0.02	30.61 ± 1.50	6.32 ± 0.35	ND	NA	NA
A-23	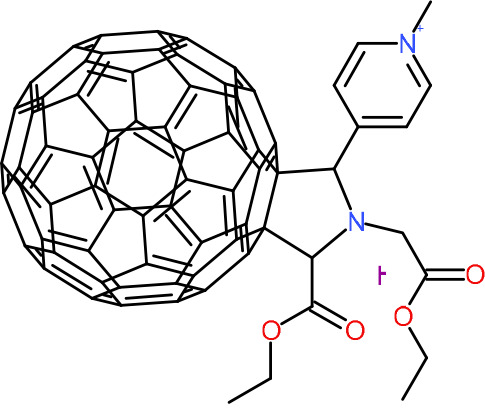	3.31 ± 0.02	4.47 ± 0.12	7.42 ± 0.46	ND	NA	NA
A-25	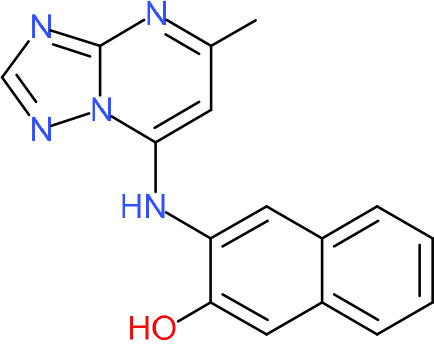	3.45 ± 0.07	1.64 ± 0.03	>50	ND	NA	NA
A-26	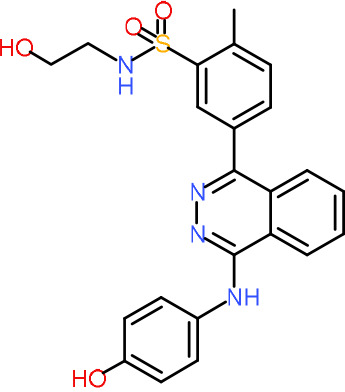	3.50 ± 0.04	14.45 ± 0.08	3.41 ± 1.22	ND	NA	NA
A-30	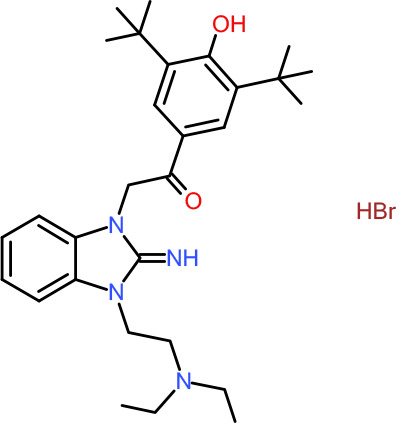	3.83 ± 0.03	0.08 ± 0.01	0.09 ± 0.06	3.75 ± 3.55	45.1	43.5
A-31	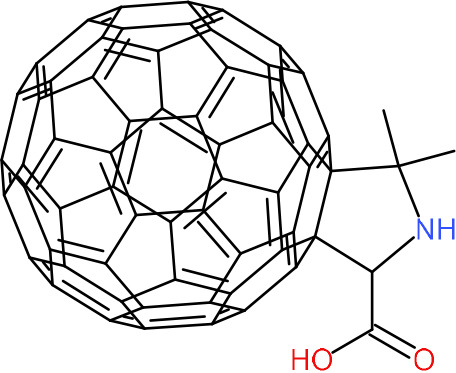	3.83 ± 0.04	>50	0.67 ± 0.02	ND	NA	NA
A-38	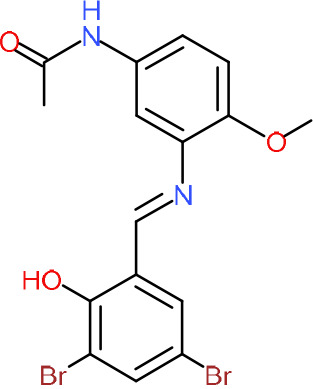	4.87 ± 0.05	29.06 ± 0.20	0.70 ± 0.10	>100	>3.4	>142.8
A-44	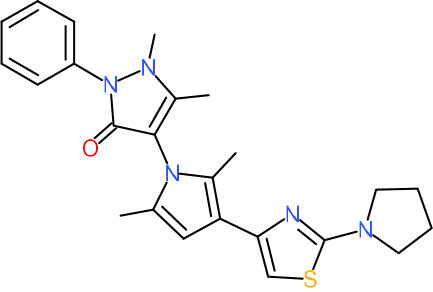	6.33 ± 0.11	3.96 ± 0.04	1.75 ± 0.05	ND	NA	NA
A-45	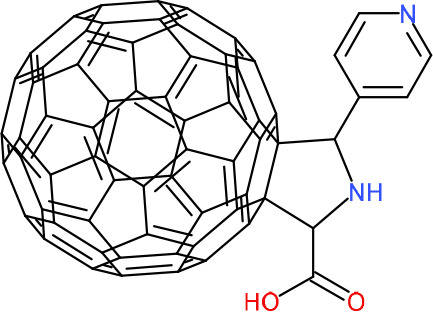	6.68 ± 0.24	15.63 ± 0.61	1.29 ± 0.02	ND	NA	NA
A-50	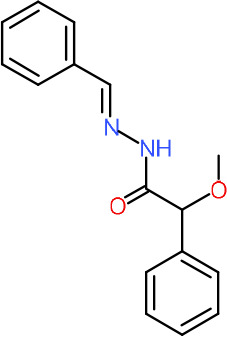	6.99 ± 0.13	>50	6.62 ± 1.12	ND	NA	NA
A-51	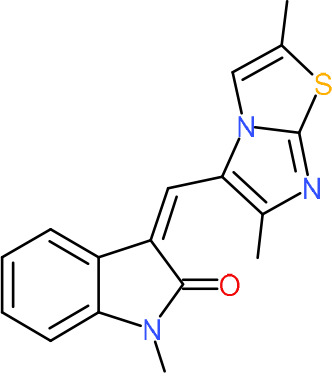	7.05 ± 0.08	15.76 ± 0.12	0.62 ± 0.23	>100	>6.3	>166.6
A-60	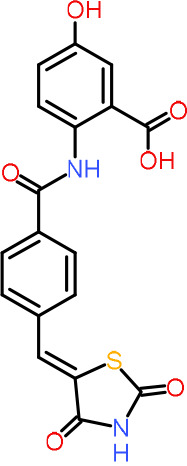	8.35 ± 0.05	34.14 ± 0.04	5.53 ± 0.95	ND	NA	NA
A-63	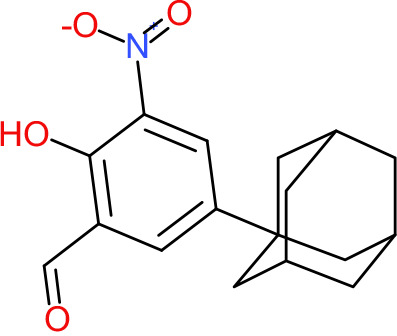	8.69 ± 0.01	6.04 ± 0.05	0.70 ± 0.22	>100	>16.6	>142.8
A-65	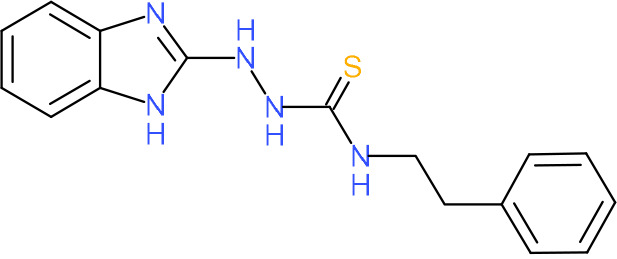	9.21 ± 0.12	4.40 ± 0.05	0.36 ± 0.10	ND	NA	NA
Atovaquone	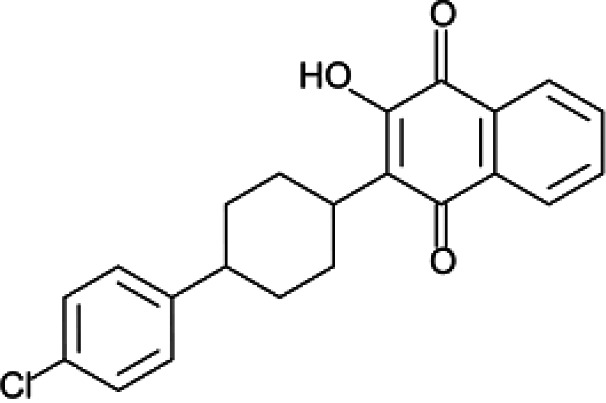	ND	0.22 ± 0.03 nM	0.16 ± 0.028 nM	79.90 ± 1.79	363,181	499,375
Mefloquine	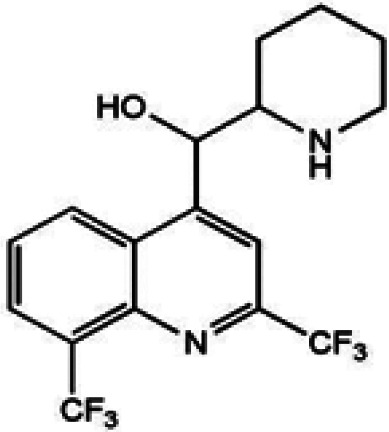	ND	0.013 ± 0.006	0.19 ± 0.07	1.73 ± 0.7	133	8.8

a Atovacuone and mefloquine were included as anti-*Plasmodium* drug control. ND, no inhibition detected in our maximum concentration tested and considered not toxic; NA, not applicable. The assays were carried out three times independently, and the results are shown as means ± SEMs of triplicates.

b Values represent micromolar unless otherwise specified.

One hundred twenty-seven potential *Pf*DPCK inhibitors were manually categorized into eight groups based on structural similarity (i.e., common scaffolds) by manual inspection. The eight groups are composed of 2 large groups, Gr1 and Gr2, with 13 and 9 hit members, respectively; 6 small groups (Gr3 to Gr8) with 2 to 6 hit members; and 87 singletons (Fig. S5). We selected Gr4, Gr5, and Gr8 as the potent *Pf*DPCK-inhibitory scaffolds based on their overall *Pf*DPCK and cell growth-inhibitory activities. Table 2 shows representative structures of these 3 groups. Especially Gr4 may represent a novel *Pf*DPCK inhibitory scaffold because it inhibited both *Pf*DPCK and P. falciparum cells with comparable IC_50_ values, and it did not show toxicity toward HepG2. However, further structure-activity relationship (SAR) studies are needed to validate the scaffold as a reasonable initial *Pf*DPCK inhibitor candidate. Regarding Gr2, it showed reasonable potency against *Pf*DPCK and P. falciparum cells, but it was excluded in the downstream analysis because of the poor physicochemical properties (e.g., high molecular weight, complex structure, and low solubility).

**TABLE 2:** Structure-activity relationship of *Pf*DPCK potent inhibitor of hit compounds

Scaffold	Compound ID	Structure	R_1_	R_2_	R_3_	IC_50_ (μM)
Gr4 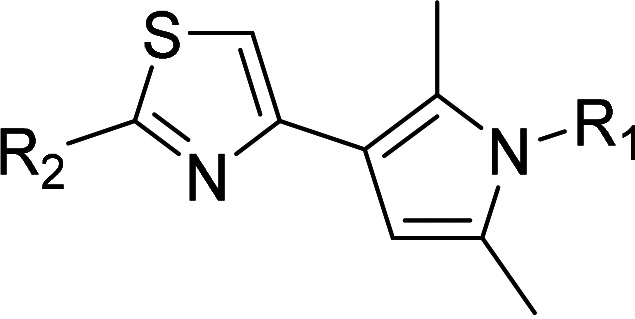	A-4	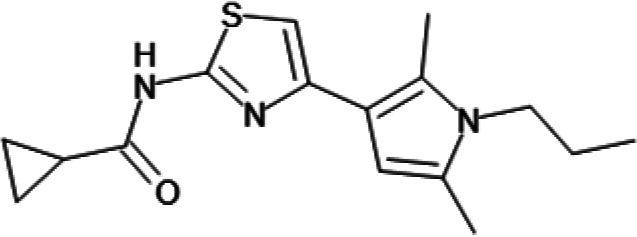	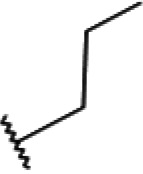	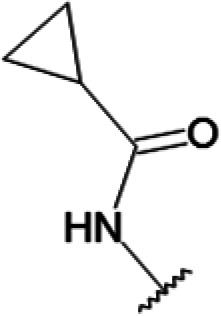		0.52 ± 0.09
	A-19	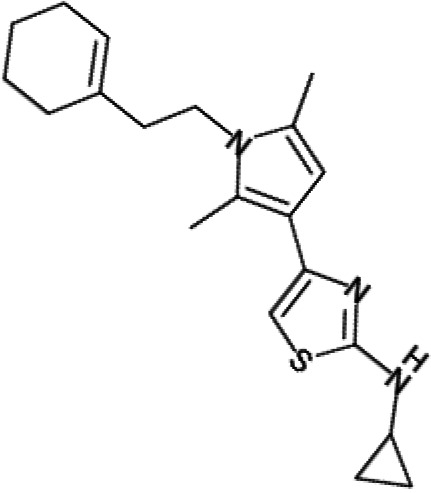	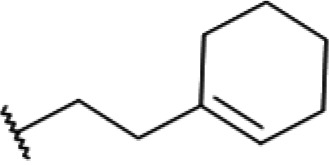	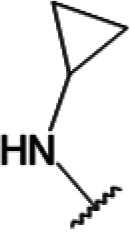		2.55 ± 0.03
	A-44	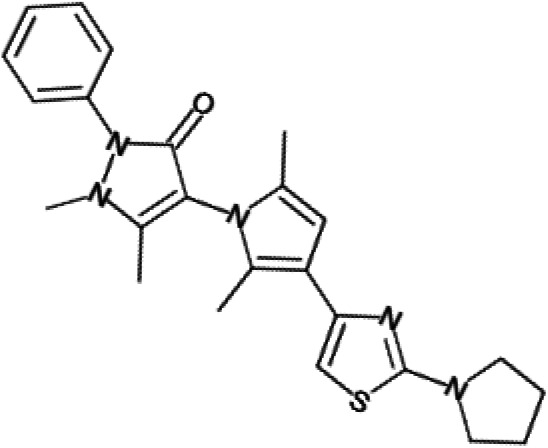	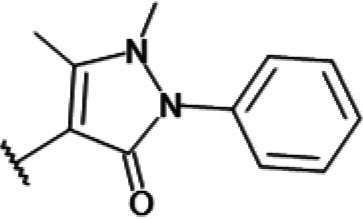	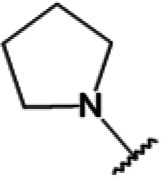		6.33 ± 0.11
	A-96	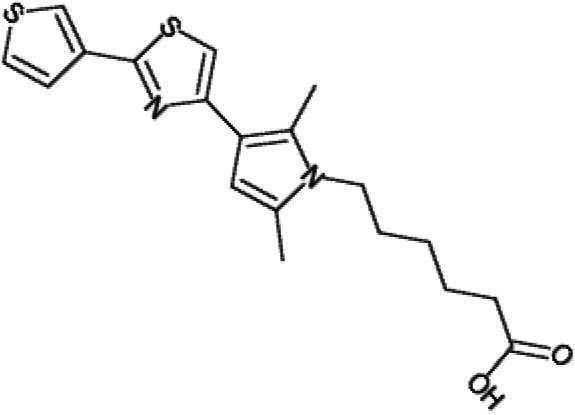	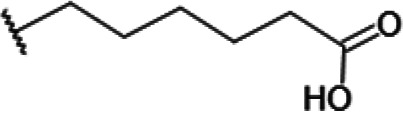	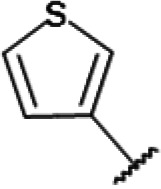		14.7 ± 0.28
Gr5 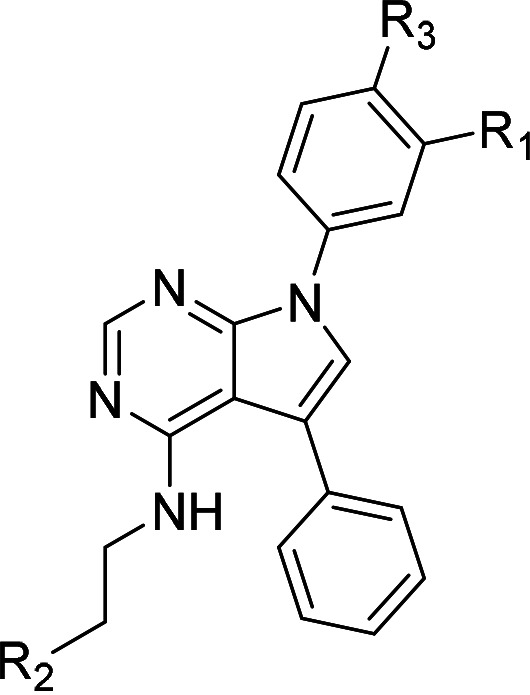	A-17	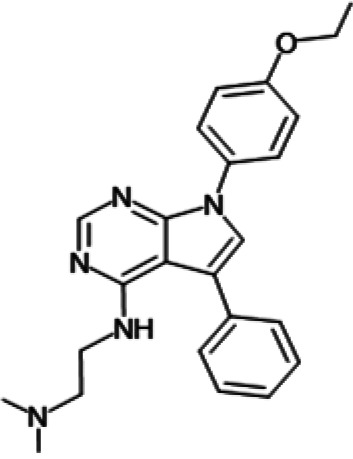	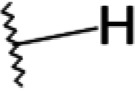	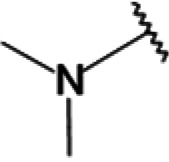	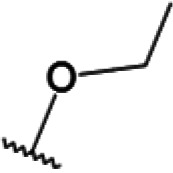	2.15 ± 0.07
	A-42	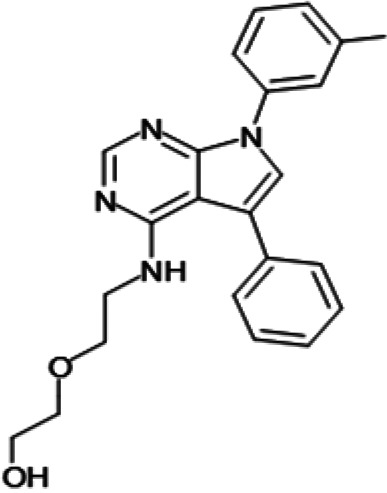	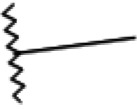	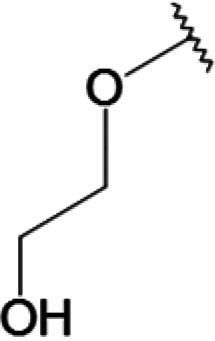	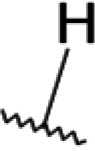	6.14 ± 0.28
Gr8 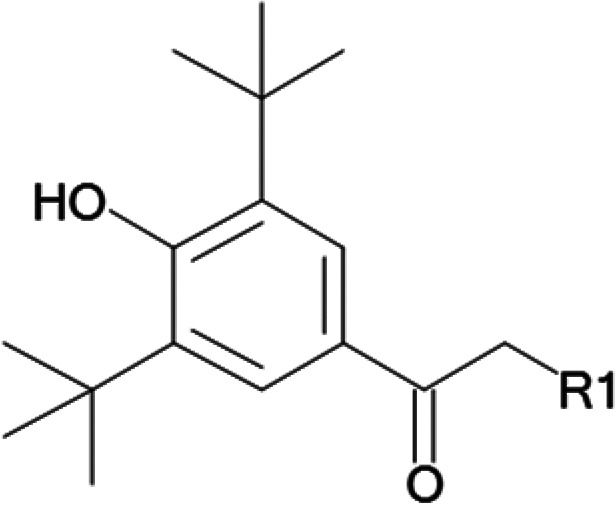	A-15	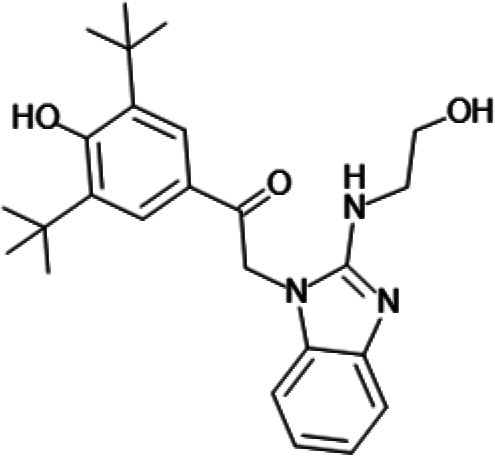	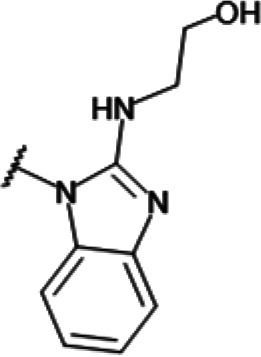			1.93 ± 0.04
	A-30	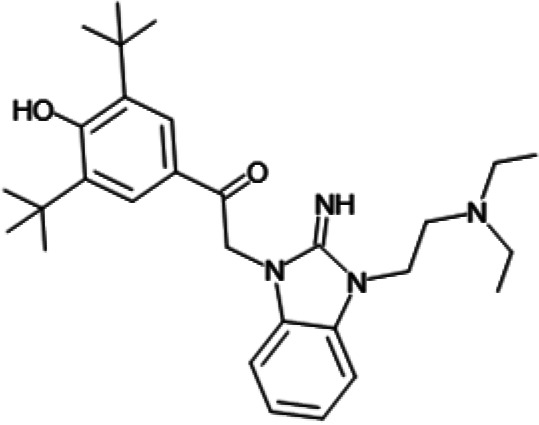	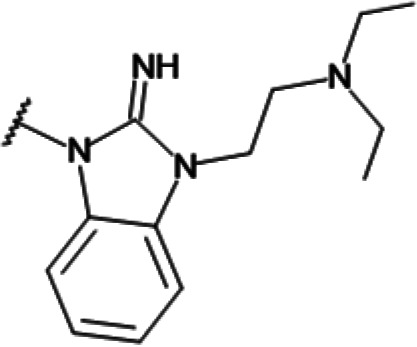			3.83 ± 0.03

### Identification and prioritization of malaria-specific DPCK inhibitors and elucidation of their mode of inhibition.

**FIG 5:**
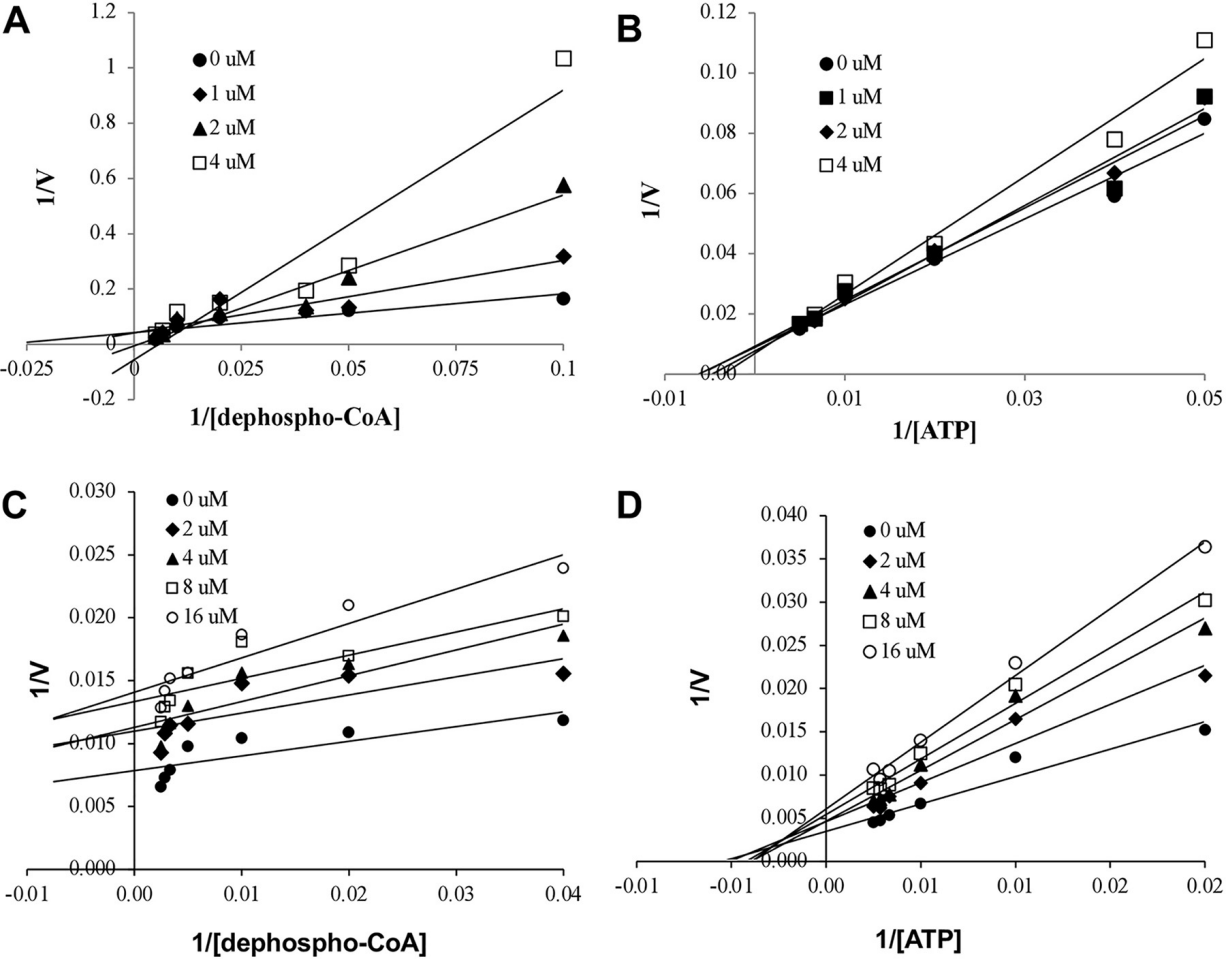
Lineweaver-Burk plots showing inhibition of two compounds, compound A-15 (A, B) and compound A-127 (C, D) on the *Pf*DPCK activity. The enzymatic activities were determined with various concentrations of dephospho-CoA and 200 μM ATP (A, C) or various concentrations of ATP and 200 μM dephospho-CoA (B, D) in the presence of a range of concentrations of inhibitors. Data are shown in means ± SEMs of triplicate.

**FIG 6:**
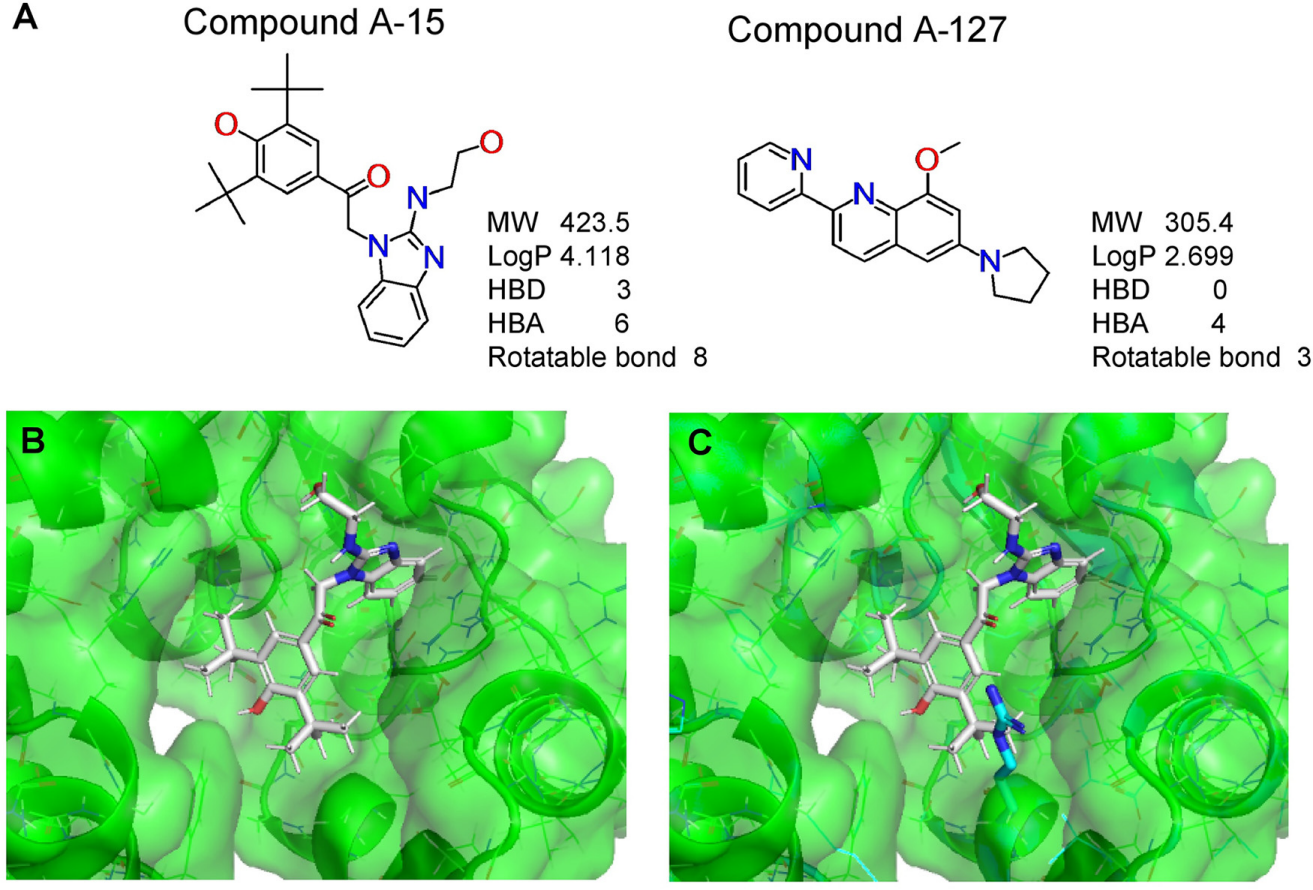
Structure of the representative *Pf*DPCK inhibitors (A) and docking simulation of compound A-15 (B, C). (A) Structure of the representative *Pf*DPCK inhibitors A-15 and A-127 and their calculated physicochemical properties. MW, molecular weight; log*P*, partition coefficient; HBD, hydrogen bond donor; HBA, hydrogen bond acceptor. (B, C) Docking simulation of compound A-15 to the catalytic pocket of *Pf*DPCK (B) and *Hs*COASY (C). The three-dimensional (3D) docking poses of compound A-15 (white sticks; oxygens are shown in red, and nitrogens are shown in dark blue) in the active sites of *Pf*DPCK (B) and *Hs*COASY (C) are shown. Key amino acid residues were determined in the 3D position in active sites. (B) The binding pose of compound A-15 with the best docking score to *Pf*DPCK is shown. (C) The structure of *Hs*COASY is superimposed with Arg514 depicted with cyan sticks, showing structural hindrance with compound A-15.

In order to understand the mechanism of *Pf*DPCK inhibition of the identified compounds, we independently selected compounds from 359 *Pf*DPCK-specific inhibitor candidates (showing >40% inhibition at 10 μM against *Pf*DPCK), based on the more stringent criteria of >80% inhibition at 10 μM against *Pf*DPCK and <10% inhibition against *Hs*COASY, to yield 30 hits. The hits were prioritized using a multiparameter optimization (MPO) scoring model, developed by MMV (https://www.optibrium.com/downloads/scoring-profiles/mmv-antimalarial-scoring-profile/) based on calculated physicochemical properties, including molecular weight, log*P*, hydrogen bond donors and acceptors, rotatable bonds, and structural alerts (20, 21). We finally chose six representative compounds (A-15, A-33, and A-69, shown in Table S1, and other three compounds; A-126, A-127, and A-128) by manual inspection with “chemist eye” for chemical attractiveness (druglikeness). All six compounds except A-127 showed no growth inhibition to 3D7 at 2 μM (Table S1). Compound A-127, which is a quinoline-containing compound, showed 80% inhibition against 3D7 at 2 μM. We found that two compounds, A-126 and A-128, also inhibit many other targets than kinase, based on the previous experimental database provided by DDI, suggesting that they are nonspecific inhibitors against numerous biological targets. Furthermore, two compounds, A-33 and A-69, are considered to be pan-assay interference compounds (PAINS) (phenolic Mannich base) (22). We consequently focused on two commercially available compounds (A-15 and A-127) (Fig. 6A) and investigated their mechanism of inhibition. We differentiated the type of inhibition by Lineweaver-Burk plot with various concentrations of one substrate (dephospho-CoA or ATP), while the other substrate was given in saturating concentrations in kinetic studies (Fig. 5). We found that compound A-15 competitively inhibits *Pf*DPCK in respect of both dephospho-CoA and ATP, with *K_i_* values of 0.47 and 10.42 μM, respectively (Fig. 5A and B). On the other hand, compound A-127 showed uncompetitive or noncompetitive inhibition for dephospho-CoA and ATP substrate, with the *K_i_* values of 14.89 and 8.99 μM, respectively (Fig. 5C and D).

### Binding mode of *Pf*DPCK by compound A-15.

To better understand the mechanism of inhibition by compound A-15 at the structural level, we performed *in silico* docking of compound A-15 as a representative *Pf*DPCK competitive inhibitor. The binding pose of compound A-15 with the best docking score is shown in Fig. 6A. The *tert*-butyl group of compound A-15 was found to be in contact with the hydrophobic side chain of *Pf*DPCK Ile230. In contrast, when the structure of *Hs*COASY was superimposed, the hydrophilic side chain of *Hs*COASY Arg514 collided with the position of the *tert*-butyl group (in Fig. 6B). This difference is consistent with the experimental results with the reciprocal plot analysis showing that compound A-15 inhibits *Pf*DPCK, but not *Hs*COASY.

## DISCUSSION

Enzymes on the CoA biosynthetic pathway have been validated as antimalarial drug targets. Among the multiple enzymes involved in the pathway, *Pf*DPCK, which catalyzes the last committed step of the pathway, is a promising target for drug development because it is encoded by a single gene, expressed in all life cycle stages, including the liver and erythrocytic stages in humans and mosquitos (23, 24), proven to be essential (13) and largely different from their human counterparts. This enzyme has an N-terminal bipartite apicoplast-trafficking peptide (25) and is confirmed as an essential apicoplast-localized protein (16).

In the present study, we identified potential hits for malaria-specific inhibitors targeting the CoA biosynthetic pathway. Although inhibitors against PanK, which catalyzes the first step of the pathway, were previously identified, our current study has provided the first case in which *Pf*DPCK was exploited as the drug target, leading to the discovery of potential leads from the structurally elucidated compound library. Although the identified *Pf*DPCK inhibitors show only moderate antiproliferative activities against the erythrocytic-stage parasites at submicro- to micromolar concentrations, our discovery of the compounds that showed comparable IC_50_ and EC_50_ values against *Pf*DPCK and the parasite have provided a proof of concept for the enzyme and the pathway are exploitable for drug discovery, despite some skepticism due to the lack of correlation between antitarget (enzymatic) and antiproliferative (cellular) activity (26) in the current trends of antimalarial discovery using phenotypic cell-based screening for the primary screening in recent years (27–29). It is worth noting that among 127 *Pf*DPCK-specific (i.e., not inhibiting *Hs*COASY) inhibitors we tested against P. falciparum blood-stage parasites, only 10 compounds (8%) show inhibition against both P. falciparum drug-sensitive 3D7 and drug-resistant Dd2 strains (with the criteria of IC_50_ of <5 μM for both enzymatic and cellular inhibition), suggesting poor permeability or instability of most of the identified *Pf*DPCK inhibitors. Thus, further chemical modifications of the identified *Pf*DPCK inhibitors have the potential to be further optimized to yield the derivatives with improved anti-erythrocytic-stage activity.

Our second-stage cell-based screening of the hits obtained by *Pf*DPCK-selective (not inhibiting *Hs*COASY) screening yielded six candidates which have the IC_50_ values of 0.40 to 4.87 μM against *Pf*DPCK and those of 0.08 to 6.04 μM against 3D7 and/or Dd2. The most promising compounds show reasonable agreement of the IC_50_ values against *Pf*DPCK and cells (i.e., the differences in the IC_50_ values are < 5-fold, and the compounds are more potent against the enzyme than the cells); however, some compounds show marked disparity for reasons that are not yet understood. For example, compound A-30 shows 4- to 10-fold lower IC_50_ values in the cell-based assay than the enzyme-based assay. These data are consistent with the hypothesis that the target of the compounds for growth inhibition is not solely via inhibition of CoA synthesis. In addition, several compounds, including A-16, A-38, A-51, and A-63, show a marked decrease in the IC_50_ values against the drug-resistant Dd2 strain compared to the drug-sensitive 3D7 (7- to 42-fold), suggesting that the resistance to one agent may lead to increased susceptibility to another. It was previously shown (30) that the development of resistance to one line of antimalarials is accompanied by the loss of resistance to other drugs. Our observation on the increased sensitivity against A-16, A-38, A-51, and A-63 in chloroquine-resistant Dd2 may also indicate that a similar interconnection of drug action may occur. In contrast, in a reverse trend, an increase in resistance was seen in Dd2 for compound A-25, suggestive of shared mechanisms of resistance (Table 1).

We also selected 30 inhibitors exclusively by virtue of selective inhibition toward *Pf*DPCK over *Hs*COASY. Among the 30 compounds, only 16 are included in the 127 compounds selected as described above. A multiparameter optimization (MPO) scoring profile was used to prioritize six compounds. The MPO was developed by MMV and allows us to rank compounds based on physicochemical properties, including molecular weight, log*P*, hydrogen bond acceptors and donors, and rotatable bonds, as well as structural alert and malaria drug fragments (21). Two representative *Pf*DPCK-specific inhibitors identified from our high-throughput screening (HTS) demonstrated a distinct mechanism of inhibition as suggested by Lineweaver-Burk plot analysis: compound A-15 displayed competitive inhibition with both of two substrates, ATP and dephospho-CoA. The selectivity for *Pf*DPCK over the human orthologue, *Hs*COASY, was rationalized by *in silico* molecular docking showing that the *tert*-butyl group of compound A-15 collides with the hydrophilic side chain of *Hs*COASY Arg514, which substitutes the important residue for strong substrate binding for *Pf*DPCK (the hydrophobic side chain of *Pf*DPCK Ile230). Thus, our docking simulation provides structural basis of the specificity of compound A-15 toward *Pf*DPCK, but not its human counterpart.

Finally, our discovery of *Pf*DPCK-specific inhibitors targeting the CoA biosynthetic pathway should provide a new validated and exploitable metabolic target that has a potential to be further pursued in the antimalarial drug development pipeline. DPCK-targeting drugs can be used in combination with existing antimalarials or new drug candidates in preclinical development or clinical trials, such as a variety of drug candidates targeting dihydroorotate dehydrogenase (DHODH); ATP4; phosphatidylinositol-4 kinase; elongation factor; acetyl-CoA synthetase; lysine-, proline-, tyrosine, and phenylalanine tRNA synthetases; proteasome; phosphodiesterase (PDE); and plasmepsin (5, 31–33).

### Conclusion.

We have provided a proof of concept that inhibitors of *Pf*DPCK can be identified from a target-based high-throughput screen. In addition, potent *Pf*DPCK inhibitors that also inhibit parasite growth in a phenotypic whole-cell asexual blood-stage assay were identified. Furthermore, the structural basis of *Pf*DPCK-specific inhibition was elucidated by a docking simulation. Although further optimization of the hits that were identified is necessary to improve efficacy, drug metabolism and pharmacokinetics (DMPK), and safety required for development as potential antimalarials, the inhibitors discovered in this study can be used as pharmacological tools for further target validation.

## MATERIALS AND METHODS

### Chemicals, microplates, organisms, and cultivation.

All chemicals of analytical grade were purchased from Sigma-Aldrich (St. Louis, MO) unless otherwise stated. ADP-hexokinase from Thermococcus litoralis (52.6 U/mg solid) was purchased from Asahi Kasei Pharma (Tokyo, Japan). Diaphorase I from Bacillus stearothermophilus (1.8 kU/mg protein) was purchased from Nipro (Osaka, Japan). Recombinant glucose-6-phosphate dehydrogenase from *Leuconostoc* sp. (G6PDH; 754 U/mg protein) and NADP^+^ were obtained from Oriental Yeast Co. (Tokyo, Japan). Triton X-100 and Tween 20 were purchased from Alfa Aesar (Lancashire, UK) and Tokyo Chemical Industry (Tokyo, Japan). *N*-ethylmaleimide (NEM), dithiothreitol (DTT), and bovine serum albumin (BSA) were purchased from Fujifilm Wako Pure Chemical (Osaka, Japan). The 1,536-well polypropylene microplates used for the kinase assay and 384-well microplates for the malaria growth inhibition assay were purchased from Greiner Bio-One (Frickenhausen, Germany).

The Plasmodium falciparum drug-sensitive 3D7 strain (GL Clone) MRA-1001 (BEI Resources, NIAID, NIH) and drug-resistant Dd2 strain were used for asexual blood-stage phenotypic assay. The cytotoxicity assay was conducted using the human hepatocarcinoma HepG2 cell line. AlbuMax II, RPMI 1640 medium, and gentamicin were purchased from Gibco (Life Technologies, Carlsbad, CA, USA); hypoxanthine was purchased from Sigma, sodium l-lactate and nitro-tetrazolium blue chloride (NBT) were purchased from Fujifilm (Wako), and 3-acetylpyridine adenine dinucleotide (APAD) was from Oriental Yeast (Japan). Dulbecco’s modified Eagle’s medium, low glucose (d-MED; with 1,000 mg/L glucose), and fetal bovine serum were purchased from Sigma-Aldrich. Trypsin-EDTA was purchased from Gibco. The overexpression of protein recombinant harboring Escherichia coli BL21(DE3) was purchased from Invitrogen (Carlsbad, CA, USA). Purification of recombinant protein used Ni^2+^-nitrilotriacetic acid (NTA) agarose, purchased from Novagen (Darmstadt, Germany). All other chemicals were analytical grade, purchased from Sigma-Aldrich (Tokyo, Japan) unless otherwise stated.

### Phylogenetic analyses of P. falciparum DPCK.

We collected 43 DPCK protein sequences from representative taxa by BLASTp search using the *Pf*DPCK protein sequence (PlasmoDB ID PF3D7_1443700; GenPept accession no. XP_001348589) as a query and nonredundant (nr) protein sequences database of National Center for Biotechnology Information (NCBI; http://www.ncbi.nlm.nih.gov/). Only protein sequences with an E value of 1 × 10^−10^ or less were selected. Sequences were aligned using the MUSCLE program (34) in SeaView package version 4.6.1 (35). The data matrices for phylogeny were subjected to the IQ-TREE program (36). The maximum-likelihood (ML) analysis implemented in the RAxML program version 7.2.6 (37) was used to infer ML tree. Trees were constructed using FigTree program version 1.4.2 (http://tree.bio.ed.ac.uk/software/figtree/). Bootstrap values higher than 50 are indicated on the corresponding internal branches of the ML tree.

### Expression and purification of recombinant *Pf*DPCK and *Hs*COASY.

The protein-coding sequences of the codon-optimized *PfDPCK* and *HsCOASY* genes were inserted into BamHI and SalI sites of the plasmid pCold1 His tag vector (TaKaRa) to produce pCold-*Pf*DPCK and pCold-*Hs*COASY. Escherichia coli BL21Star (DE3) chemically competent cells (Thermo Fisher Scientific, Waltham, MA, USA) were transformed with these plasmids and cultured at 37°C in 500 mL of Luria-Bertani medium (LB, Invitrogen) in the presence of 100 μg/mL ampicillin (Nacalai Tesque). The overnight culture was used to inoculate 1 L of fresh LB medium. The culture was continued at 37°C with shaking at 180 rpm until the *A*_600_ reached 0.8. After isopropyl β-d-thiogalactopyranoside (IPTG) was added at a final concentration of 0.5 mM, cultivation was continued for another 24 h at 15°C. Cultured E. coli cells were harvested by centrifugation at 5,000 × *g* for 20 min at 4°C. The cell pellet was collected and resuspended in 40 mL of the lysis buffer (50 mM Tris-HCl, pH 8.0, 300 mM NaCl, and 10 mM imidazole) containing 0.1% Triton X-100 (vol/vol), 0.7 M trehalose, 100 μg/mL lysozyme, and 1 mM phenylmethylsulfonyl fluoride (PMSF) and incubated at room temperature for 30 min. The cells were then passed through a French press (Ohtake, Tokyo) with a pressure of 800 kg/cm^2^, and the lysate was centrifuged at 25,000 × *g* for 30 min at 4°C to remove debris. The supernatant obtained was mixed with 2 mL of 50% Ni^2+^-NTA His-bind slurry (Qiagen, Germany) and then incubated at 4°C with mild shaking for 1 h. The resin bound to recombinant enzymes was washed three times with washing buffer (50 mM Tris-HCl, pH 8.0, and 300 mM NaCl, containing 20 mM imidazole and 0.1% [vol/vol] Triton X-100). Bound enzymes were eluted with washing buffer containing stepwise gradient concentrations of imidazole (20 to 300 mM). The purity of recombinant protein was confirmed by 12% SDS-PAGE analysis, followed by Coomassie brilliant blue (CBB) staining. Purified proteins were dialyzed against a 300-fold volume of dialysis buffer (50 mM Tris-HCl, pH 8.0, 150 mM NaCl, containing 10% glycerol [vol/vol] supplemented with Complete Mini protease inhibitor cocktail [Roche, Mannheim, Germany]) at 4°C for 18 h to remove residual imidazole. Enzymes were stored at −80°C with a final 20% glycerol in small aliquots until use.

### Enzyme assays.

*Pf*DPCK and *Hs*COASY activity was measured by quantifying ADP produced in the reaction by a coupling assay using the ADP Hunter Plus assay kit (DiscoverX, USA) according to the manufacturer's instructions. Briefly, enzymatic reactions were carried out in a 20-μL mixture of assay buffer containing 15 mM HEPES, 20 mM NaCl, 1 mM EGTA, 0.02% Tween 20, 10 mM MgCl_2_, 0.1% bovine gamma globulin, 50 ng of recombinant *Pf*DPCK or *Hs*COASY, 4 to 256 μM dephospho-CoA, and 5 to 300 μM ATP on a black microplate. All reactions were performed for 2 h, reagents A and B were added, and they were then reincubated for 60 min at 30°C. After reactions were terminated by addition of ADP Hunter Stop solution, the fluorescent signal was measured using SpectraMax Paradigm (Molecular Devices, CA, USA) at excitation and emission wavelengths of 530 and 590 nm, respectively. The kinetic parameters were calculated using the nonlinear regression function using the single saturating concentrations of the substrates by the GraphPad Prism software (GraphPad Software Inc., San Diego, CA). The experiments were repeated three times in triplicate using proteins purified from independent cultures. Kinetic values of enzymes are presented as the means ± standard errors (SEs).

### High-throughput screening of structurally diversified chemical library.

High-throughput screening against recombinant *Pf*DPCK was performed using the Drug Discovery Initiative (DDI) full library, available from The University of Tokyo, composed of approximately 210,000 compounds. The primary assay was performed on 1,536-well white plate format with a final volume of 2 μL/well, containing 10 μM test compounds using a Multidrop Combi reagent dispenser (Thermo Fisher Scientific, Waltham, MA). The reaction was conducted in the assay buffer described above with 100 μM ATP and 50 μM dephospho-CoA. The assay was carried out at room temperature for 4 h in a closed plastic container with wet tissues. The reaction was stopped, and produced ADP was quantified using an enzyme-coupled fluorescence ADP detection assay, using PHERAstar microplate reader (BMG Labtech, Offenburg, Germany) with excitation at 540 nm and emission at 590 nm, as previously described (17). The inhibition level was calculated as the percentage inhibition of DPCK activity with each compound compared to the activity with only dimethyl sulfoxide (DMSO) as control (no inhibition) and that with no enzyme as 100% inhibition. IC_50_ values of the hit compounds were determined under the same assay conditions for the screening: Z′ factor, signal-to-background ratio (S/B), signal-to-noise ratio (S/N), and coefficient of variation (CV) were calculated to estimate the quality of screening system as previously described (18, 38).

### Reconfirmation, counterassays, and IC_50_ determination of *Pf*DPCK inhibitors.

Compounds that showed >40% inhibition against *Pf*DPCK at 10 μM in the primary assay were selected for secondary reconfirmation assay. The primary hits were reassayed in quadruplicate (*n* = 4) as in the primary screening. We next tested the confirmed secondary hits to exclude false positives that inhibit coupled fluorescence assay, but not *Pf*DPCK. After exclusion of false positives, all tertiary hits were tested against human *Hs*COASY in the counterassay to exclude compounds that inhibit both *Pf*DPCK and *Hs*COASY. The compounds showing >10% inhibition against *Hs*COASY were excluded from further studies. The IC_50_ values of the hit compounds were determined under the same assay conditions described as above for the primary screening, with the serially diluted compounds at 20, 10, 2.5, 0.5, and 0.1 μM at final concentrations. The assay was conducted in quadruplicate for each dilution, and IC_50_ values were calculated using GraphPad Software (San Diego, California USA).

### Estimation of IC_50_ values against P. falciparum 3D7 erythrocytic-stage parasite growth.

Selected hit compounds were also tested against erythrocytic-stage parasites of P. falciparum drug-sensitive 3D7 and drug-resistant Dd2 strains (39). For the phenotypic assay, parasite cultures were synchronized with 5% (wt/vol) d-sorbitol as previously described (40). A synchronized culture containing ring-stage parasites of 0.3% parasitemia was initiated with a culture volume of 25 μL/well on a 384-well plate. A series of diluted compounds (at final concentrations of 50, 10, 2, 0.4, and 0.08 μM) were used to calculate IC_50_ values. Mefloquine at 50 μM and atovaquone at 20 μM were used as no-growth control (100% inhibition). After 72 h of incubation, parasite growth was measured by diaphorase-coupled lactate dehydrogenase (LDH) assay as previously described (41). Absorbance at 650 nm was measured using SpectraMax Paradigm multimode microplate reader (Molecular Devices, San Jose, CA, USA). The IC_50_ values were calculated as described above.

### Cytotoxicity assay.

Cytotoxicity against human cells was evaluated using the human liver carcinoma HepG2 cell line. HepG2 cells were cultured in d-MED supplemented with 4 mM l-glutamine and 10% fetal bovine serum (FBS) in a 75-cm^2^ flask (Violamo) at 37°C. For the cytotoxicity assay, HepG2 cells in semiconfluent state were detached from the flask by incubating the cells with 5 mL d-MEM containing 0.25% trypsin-EDTA. After detachment, HepG2 cells were resuspended in d-MEM-supplemented l-glutamine and FBS, and cell viability was evaluated by incubating cells with 0.4% Trypan blue on a hemocytometer. An approximately 50-μL suspension containing 3,000 cells was dispensed into each well on a 384-well clear-bottom plate using a Multidrop Combi dispenser. Compounds dissolved in DMSO had been dispensed into wells to yield final concentrations of 100, 20, 4, 0.8, and 0.2 μM before cells were added. After cells were added to the plates, they were cultured at 37°C under 5% CO_2_. After 48 h cultivation, 5 μL of Cell Counting Kit-8 (Dojindo, Japan) was added, and the plates were further incubated for 2 h to assess cell growth and survival. The absorbance wavelengths of 450 nm were measured on the microplate reader, and the IC_50_ values were calculated as above.

### Creation of protein structure models for docking simulation.

Since the crystal structure of *Pf*DPCK is not available in the public database and our repeated attempts to make a crystal were unsuccessful, we *in silico* predicted the structure of *Pf*DPCK and *Hs*COASY by AlphaFold2 (42, 43) (see Fig. S2A in the supplemental material). The predicted structure contains three potential membrane-bound helices in the N terminus (Fig. S2B and S2C). The first membrane-associated alpha helixes from the N terminus are predicted to be a part of signal peptide- and apicoplast-targeting sequence (the amino acid sequence MFLKFFLDKCILCFLAL^−^). The organisms that *Pf*DPCK show the highest similarity to are DPCK from prokaryotes. The superposition of *Pf*DPCK and DPCK from Mycobacterium paratuberculosis, Sulfolobus solfataricus, and Campylobacter jejuni is shown in Fig. S3. The predicted protein structures were obtained from AlphaFold Protein Structure Database (42), with the database entry IDs of Q8IL34 and Q13057 for *Pf*DPCK and *Hs*COASY, respectively. The predicted structure of *Hs*COASY shows a closed conformation of the active site that has contact with the substrates, while the predicted structure of *Pf*DPCK shows an open conformation without the substrate (Fig. S4A). To better align the backbone of *Pf*DPCK with that of *Hs*COASY, linear morphing was applied using PyMOL (version 2.4.1) (Fig. S4B).

### Prediction of the binding of *Pf*DPCK and its inhibitor by docking simulation.

The *Pf*DPCK and *Hs*COASY structures prepared above were used for docking simulation. The three-dimensional (3D) structure of compound A-15 (registered as CID 2852293) was obtained from PubChem https://pubchem.ncbi.nlm.nih.gov/compound/2852293. The docking simulation was performed using Molegro Virtual Docker (version 7.0.0) (44). We defined the search space as a sphere with a radius of 8 Å centered on the middle region of the binding sites of ATP and dephospho-CoA (Fig. S4C), considering the experimental results of the inhibition mode. Compound A-15 was docked with the default settings of the software with the following modifications: scoring function, PLANTS score (GRID); search algorithm, GPU screening with energy minimization.
