# Supplementary material for: Dephospho-Coenzyme A Kinase Is an Exploitable Drug Target against Plasmodium falciparum: Identification of Selective Inhibitors by High-Throughput Screening of a Large Chemical Compound Library
Source: Antimicrob Agents Chemother. 2022 Oct 31;66(11):e00420-22. doi: 10.1128/aac.00420-22 (PMC9664868; doi:10.1128/aac.00420-22)
Supplement: Supplemental file 1 — Supplemental material. Download aac.00420-22-s0001.pdf, PDF file, 2.5 MB [file aac.00420-22-s0001.pdf]

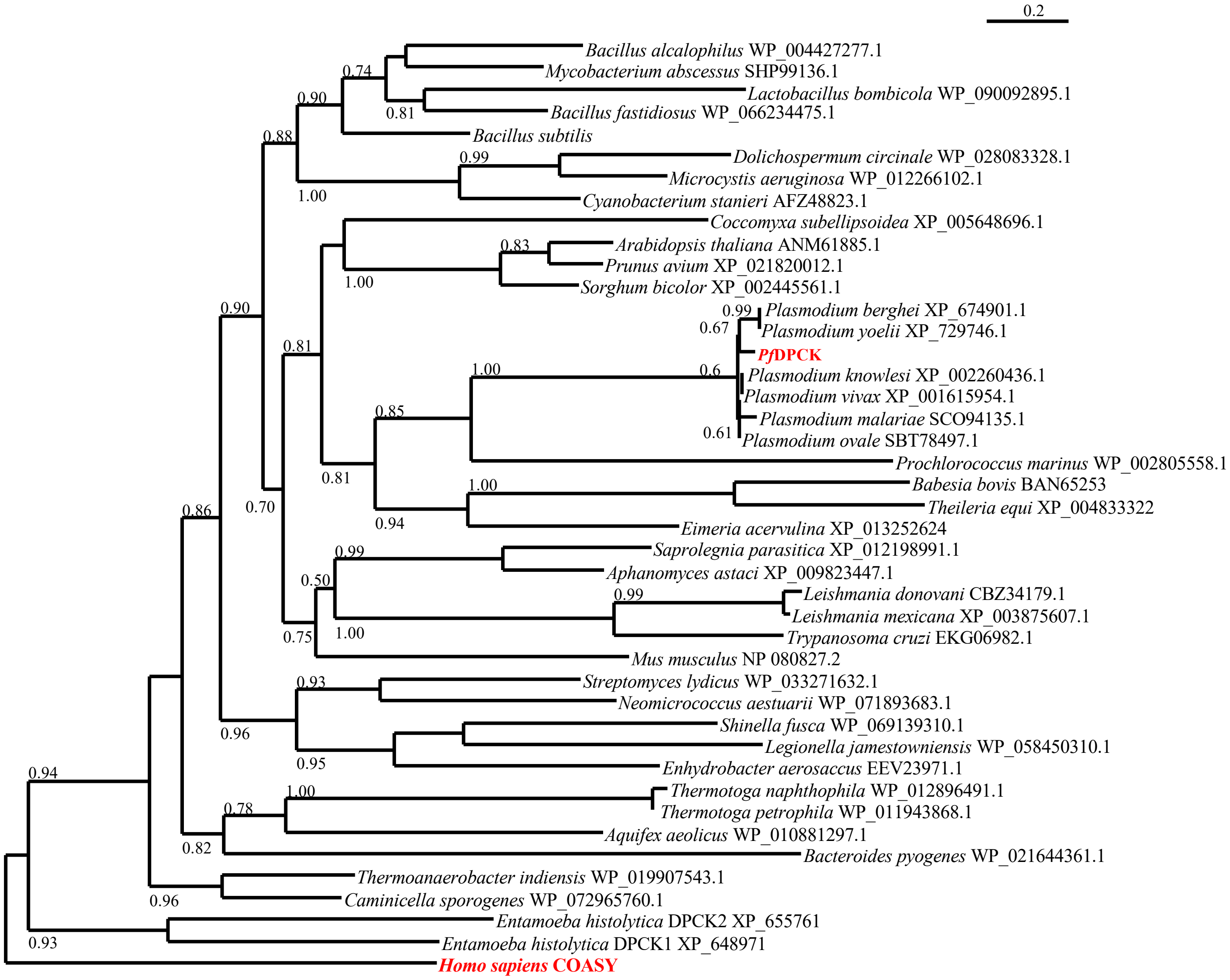

Figure S1. Phylogenetic analysis of DPCK protein sequences from *P. falciparum* and other organisms. Optimal ML tree inferred by RAxML program with LG +Γ4 model using 190 amino acid residues is shown. The numbers at the nodes represent the bootstrap values as a percentage of 100 replicates. The scale bar indicates 0.2 substitutions at each amino acid position.

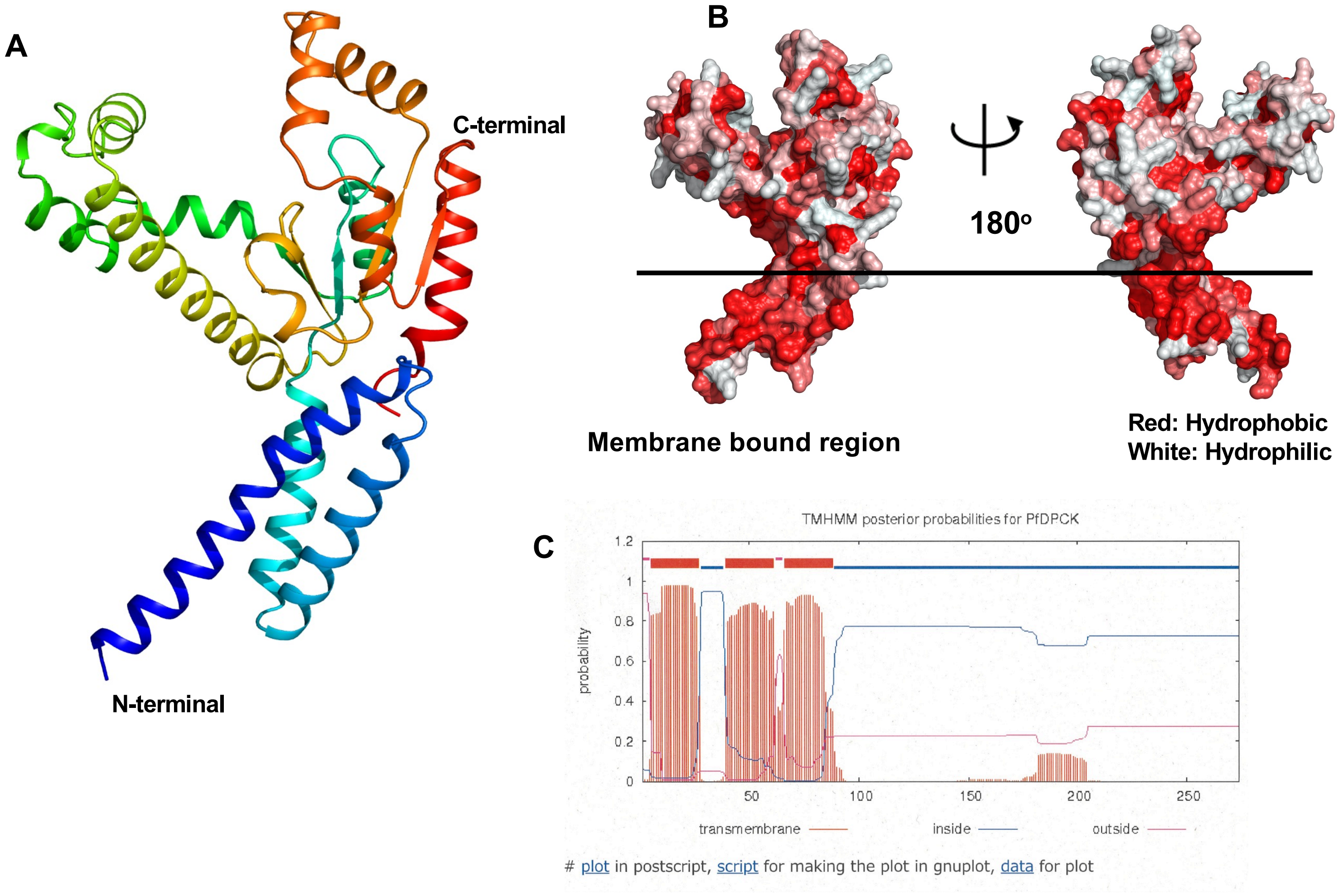

Figure S2. The three-dimensional model of the structure of *Pf*DPCK, calculated with AlphaFold2 (A) A ribbon diagram of three-dimensional structure of *Pf*DPCK in a N terminus-to-C terminus color gradient. (B) Representation of molecular surface of *Pf*DPCK in two rotated (180º) views. Red, hydrophobic region; white, hydrophilic region. (C) Three membrane bond helices are predicted in the N-terminus of PfDPCK protein. The first 17 amino acid sequence in N-terminus are predicted as apicoplast targeting peptide.

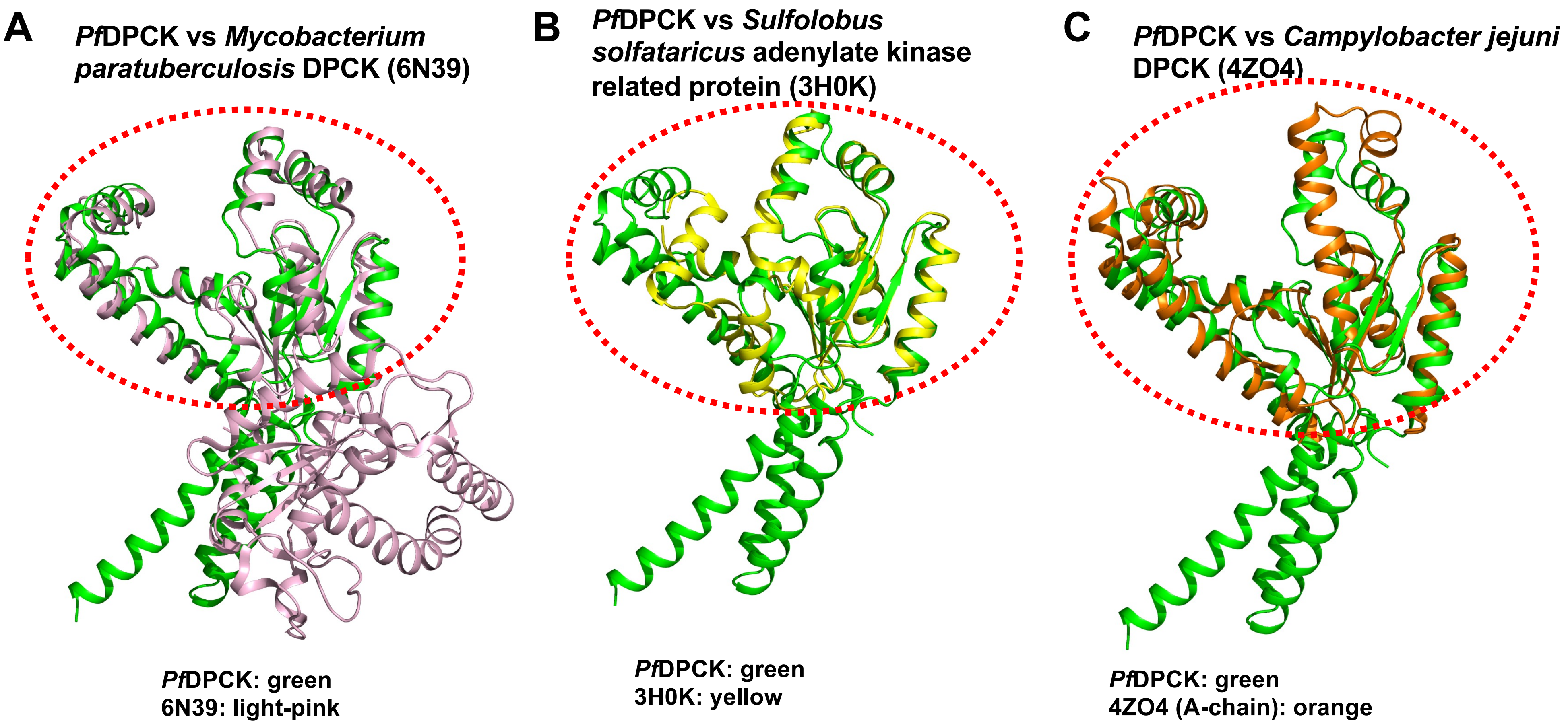

Figure S3. The comparison of the predicted structure of *Pf*DPCK with the closest orthologs from other species, DPCK from *Mycobacterium paratuberculosis* (PDB ID: 6N39) (A), adenylate kinase related protein from *Sulfolobus solfataricus* (3H0K) (B), and DPCK from *Campylobacter jejuni* (4ZO4) (C). Red dotted circles indicate the regions where two proteins are superposed well.

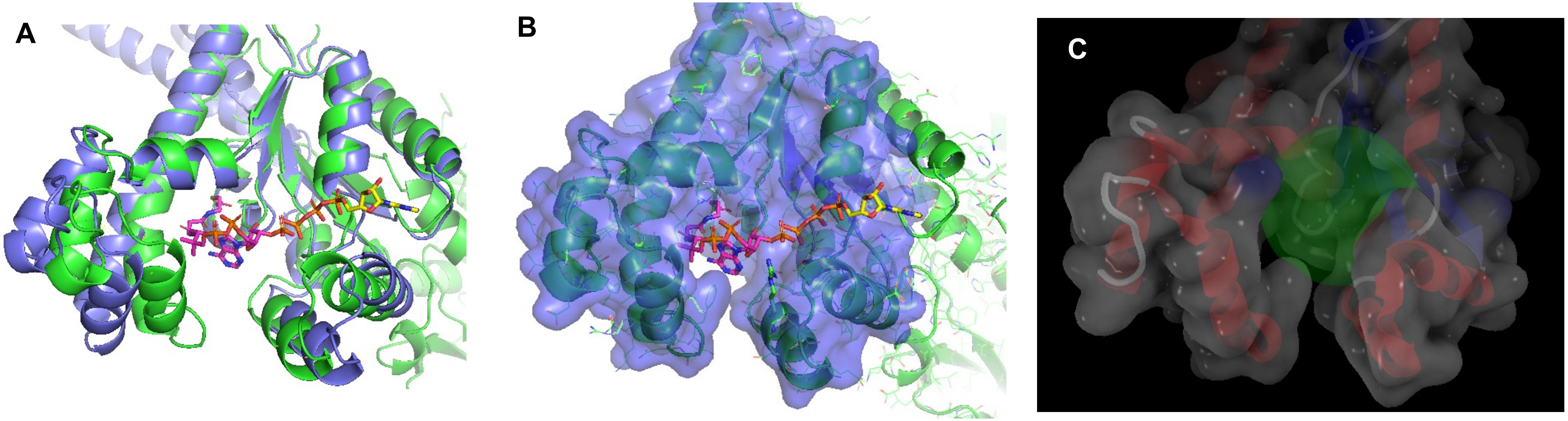

***Pf*DPCK: blue**
***Hs*COASY: green**
CoA: orange
ATP: yellow

***Pf*DPCK: blue**
***Hs*COASY: green**
CoA: orange
ATP: yellow

Figure S4. Preparation of the *Pf*DPCK structure for molecular docking. (A) Superimposition of the structures of *Pf*DPCK (blue) and HsCOASY (green) predicted by AlphaFold2. The coordinates of CoA (orange) and ATP (yellow) are adopted from 2F6R.PDB and 4TTQ.PDB, respectively. (B) The surface representation of *Pf*DPCK over the ribbon diagram shown in (A). (C) For the backbone structure alignment of *Pf*DPCK to *Hs*COASY, search space definition for docking simulation, a sphere with a radius of 8 Å is centered on the middle region of the binding site of ATP and dephosphoCoA.

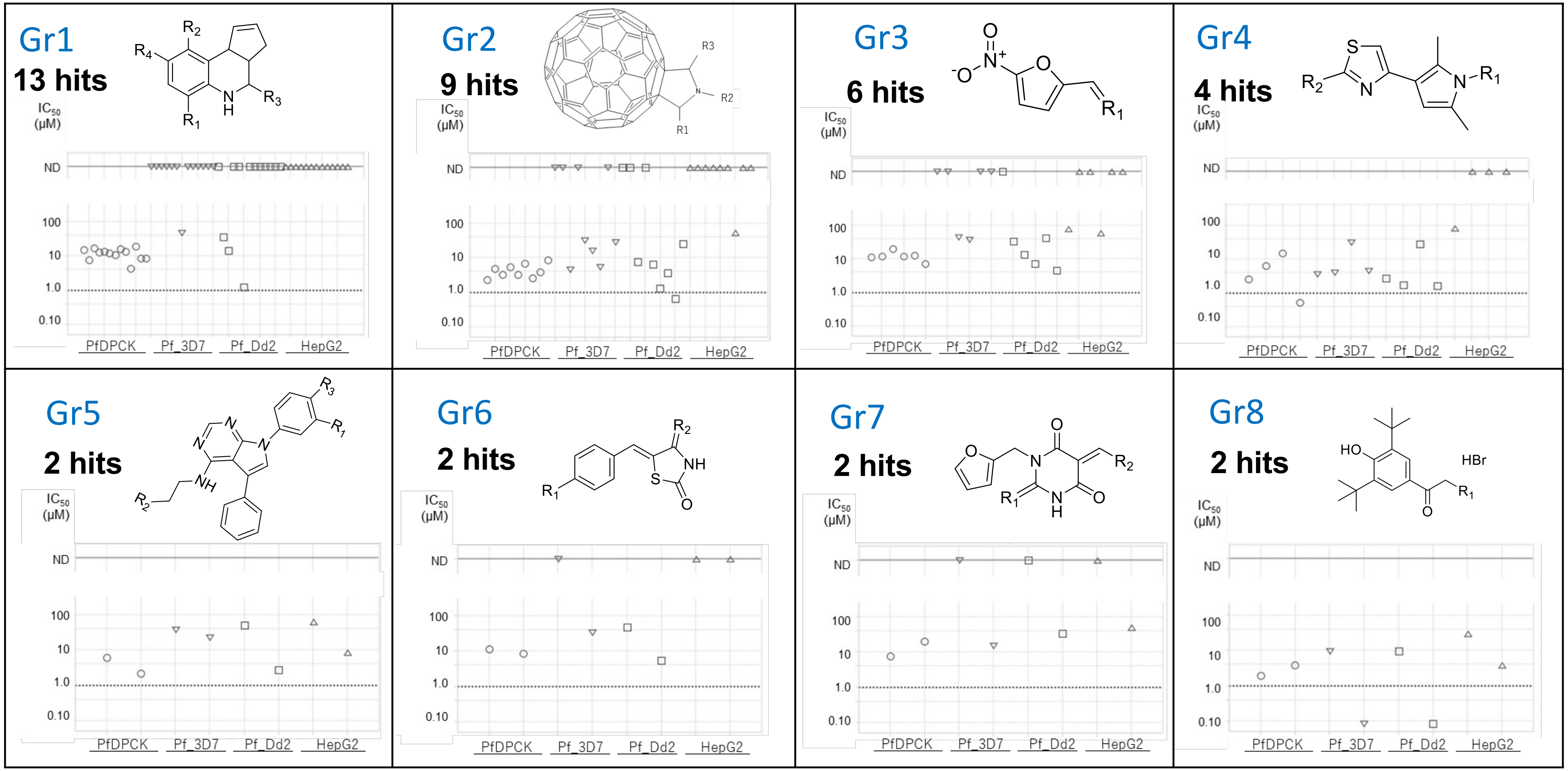

○ IC_{50} *Pf*DPCK, ▽ IC_{50} *P. falciparum* 3D7, □ IC_{50} *P. falciparum* Dd2, △IC_{50} HepG2
ND = IC_{50} is not determined (ND, >100, >50, and so on)

Figure S5. Clustering of *Pf*DPCK inhibitors from 127 hits into chemical scaffolds. The values of IC_{50} to *Pf*DPCK enzyme, *P. falciparum* cell (3D7 and Dd2) and liver human cell line (HepG2) are plotted.

Table S1. The IC50 values of total 127 compounds against *Pf*DPCK, P. falciparum erythrocytic stage cells of drug-sensitive (3D7) and resistant (Dd2) strains and human liver cell line (HepG2). The compounds showing the IC50 value of < 30 µM against *Pf*DPCK, the IC50 value of < 10 µM against either *P. falciparum* 3D7 or Dd2 strain, or both, and the EC50 value of > 100 µM against HepG2, or the selective index >10, are shown. The assays were carried out three times independently, and the results are shown as means ± SEM of triplicates.

Compound	IC_{50} (µM)				Selectivity Index (SI) 3D7 to HepG2	Selectity Index (SI) DD2 to HepG2
	Pf DPCK	Pf cell 3D7	Pf cell Dd2	HepG2		
A-1	0.36 ± 0.16	41.59 ± 2.84	ND	ND	ND	ND
A-2	0.40 ± 0.07	4.43 ± 0.04	0.98 ± 0.50	>100	>23	>102
A-3	0.51 ± 0.01	>50	ND	>100	>2	
A-4	0.52 ± 0.09	4.45 ± 0.05	1.59 ± 0.23	ND	ND	ND
A-5	0.68 ± 0.02	25.13 ± 1.73	34.04 ± 1.36	>100	>3.9	>2.9
A-7	0.05 ± 0.03	1.10 ± 0.11	0.13 ± 0.00	9.04 ± 0.10	8.2	69.5
A-8	1.12 ± 0.08	>20	>20	ND	ND	ND
A-9	1.21 ± 0.02	0.29 ± 0.02	0.18 ± 0.08	>100	>344.8	>555.5
A-10	1.30 ± 0.14	28.15 ± 1.56	2.53 ± 0.98	56.68 ± 3.56	2	22.4
A-11	1.55 ± 0.09	32.65 ± 1.91	23.43 ± 5.27	>100	>3	>4.2
A-12	1.59 ± 0.04	1.38 ± 0.14	0.20 ± 0.14	61.29 ± 1.82	44.3	306.5
A-13	1.69 ± 0.05	1.30 ± 0.06	3.40 ± 0.35	ND	ND	ND
A-14	1.88 ± 0.04	>50	49.48 ± 5.44	>100	>2	>2
A-15	1.93 ± 0.04	9.78 ± 0.23	9.26 ± 0.26	29.92 ± 3.61	3.1	3.2
A-16	2.09 ± 0.04	2.21 ± 0.07	0.23 ± 0.01	39.64 ± 2.30	17.9	171.7
A-17	2.15 ± 0.07	22.88 ± 1.87	2.70 ± 0.44	8.45 ± 0.35		
A-18	2.26 ± 0.02	>50	>50	ND	ND	ND
A-19	2.55 ± 0.03	3.64 ± 0.04	2.65 ± 0.13	81.50 ± 3.54	22.4	30.8
A-20	2.64 ± 0.01	5.39 ± 0.86	3.59 ± 0.84	53.99 ± 3.78	10.0	15.0
A-21	3.04 ± 0.05	>50	ND	>100		
A-22	3.25 ± 0.02	30.61 ± 1.50	6.32 ± 0.35	ND	ND	ND
A-23	3.31 ± 0.02	4.47 ± 0.12	7.42 ± 0.46	ND	ND	ND
A-24	3.45 ± 0.03	>50	ND	>100		
A-25	3.45 ± 0.07	1.64 ± 0.03	>50	ND	ND	ND
A-26	3.50 ± 0.04	14.45 ± 0.08	3.41 ± 1.22	ND	ND	ND
A-27	3.53 ± 0.03	>50	ND	>100		
A-28	3.74 ± 0.11	32.47 ± 1.17	10.47 ± 1.25	ND	ND	ND
A-29	3.74 ± 0.02	31.19 ± 2.65	21.58 ± 7.97	>100	>3.2	>4.6
A-30	3.83 ± 0.03	0.08 ± 0.01	0.09 ± 0.06	3.75 ± 0.40	45.1	43.5
A-31	3.83 ± 0.04	>50	0.67 ± 0.02	ND	ND	ND
A-32	3.83 ± 0.11	33.03 ± 1.30	13.72 ± 2.97	69.46 ± 1.07	2.1	5.1
A-33	4.21 ± 0.02	>50	ND	>100		
A-34	4.28 ± 0.05	>50	>50	ND	ND	ND
A-35	4.36 ± 0.01	ND	>50	ND	ND	ND
A-36	4.61 ± 0.09	>50	ND	ND	ND	ND
A-37	4.69 ± 0.04	>50	>50	ND	ND	ND
A-38	4.87 ± 0.05	29.06 ± 0.20	0.70 ± 0.10	>100	>3.4	>142.8
A-39	5.36 ± 0.05	>50	ND	ND	ND	ND
A-40	5.43 ± 0.04	>50	ND	ND	ND	ND
A-41	5.81 ± 0.12	>50	5.38 ± 0.58	80.79 ± 3.12	<1.6	15.2
A-42	6.14 ± 0.28	38.16 ± 2.91	49.72 ± 3.22	63.24 ± 3.48	1.7	1.3
A-43	6.20 ± 0.28	26.37 ± 0.01	0.54 ± 0.23	12.27 ± 0.52	0.5	22.7
A-44	6.33 ± 0.11	3.96 ± 0.04	1.75 ± 0.05	ND	ND	ND
A-45	6.68 ± 0.24	15.63 ± 0.61	1.29 ± 0.02	ND	ND	ND
A-46	6.69 ± 0.55	>50	>50	>100	>2	>2

A-47	6.70 ± 0.02	33.82 ± 5.60	ND	ND	ND	ND
A-48	6.87 ± 0.06	>50	>50	>100	>2	>2
A-49	6.88 ± 0.12	31.06 ± 0.32	10.99 ± 6.84	ND	ND	ND
A-50	6.99 ± 0.13	>50	6.62 ± 1.12	ND	ND	ND
A-51	7.05 ± 0.08	15.76 ± 0.12	0.62 ± 0.23	>100	>6.3	>166.6
A-52	7.34 ± 0.04	>50	4.66 ± 0.04	ND	ND	ND
A-53	7.34 ± 0.03	44.17 ± 3.30	19.20 ± 6.02	ND	ND	ND
A-54	7.45 ± 0.07	35.27 ± 3.46	10.07 ± 0.69	ND	ND	ND
A-55	7.59 ± 0.05	>50	>50	ND	ND	ND
A-56	7.66 ± 0.14	>20	ND	ND	ND	ND
A-57	7.75 ± 0.12	>50	34.47 ± 2.16	ND	ND	ND
A-58	8.21 ± 0.96	>50	>50	ND	ND	ND
A-59	8.30 ± 0.52	>50	>50	ND	ND	ND
A-60	8.35 ± 0.05	34.14 ± 0.04	5.53 ± 0.95	ND	ND	ND
A-61	8.61 ± 0.05	>50	ND	ND	ND	ND
A-62	8.64 ± 0.04	28.23 ± 0.22	24.64 ± 7.58	ND	ND	ND
A-63	8.69 ± 0.01	6.04 ± 0.05	0.70 ± 0.22	>100	>16.6	>142.8
A-64	9.04 ± 0.05	35.35 ± 3.25	>50	ND	ND	ND
A-65	9.21 ± 0.12	4.40 ± 0.05	0.36 ± 0.10	ND	ND	ND
A-66	9.44 ± 0.05	>50	ND	ND	ND	ND
A-67	9.78 ± 0.02	41.83 ± 5.41	19.17 ± 1.74	ND	ND	ND
A-68	10.01 ± 0.11	>50	>50	ND	ND	ND
A-69	10.11 ± 0.06	2.66 ± 1.16	ND	ND	ND	ND
A-70	10.42 ± 0.47	>50	ND	ND	ND	ND
A-71	10.48 ± 0.06	45.68 ± 0.45	>50	ND	ND	ND
A-72	11.10 ± 0.09	>50	10.00 ± 0.13	ND	ND	ND
A-73	11.36 ± 2.01	>50	46.32 ± 0.66	ND	ND	ND
A-74	11.42 ± 0.05	>50	>50	76.16 ± 4.67		
A-75	11.60 ± 0.16	31.62 ± 1.67	16.21 ± 0.14	96.75 ± 5.02	3.1	6.0
A-76	11.70 ± 0.41	>50	1.24 ± 0.09	>100	>2	>80.6
A-77	11.75 ± 0.11	>50	ND	ND	ND	ND
A-78	12.02 ± 0.18	37.55 ± 4.55	>50	ND	ND	ND
A-79	12.10 ± 0.28	>50	32.81 ± 9.63	ND	ND	ND
A-80	12.15 ± 0.05	36.73 ± 1.79	7.33 ± 3.98	59.10 ± 0.57	1.6	8.1
A-81	12.15 ± 0.43	>50	>50	ND	ND	ND
A-82	12.44 ± 0.20	>50	41.24 ± 11.18	ND	ND	ND
A-83	12.50 ± 0.08	>50	>50	ND	ND	ND
A-84	12.60 ± 0.09	53.41 ± 3.09	ND	ND	ND	ND
A-85	12.66 ± 0.11	>50	>50	ND	ND	ND
A-86	12.80 ± 0.14	>50	>50	ND	ND	ND
A-87	12.84 ± 0.46	>50	ND	>100		
A-88	12.95 ± 0.17	30.27 ± 0.15	8.95 ± 1.69	41.06 ± 1.14	1.4	4.6
A-89	13.42 ± 0.52	>50	ND	ND	ND	ND
A-90	13.61 ± 0.12	4.43 ± 0.05	4.80 ± 1.56	37.50 ± 7.68	8.5	7.8
A-91	13.63 ± 0.14	>50	ND	ND	ND	
A-92	13.65 ± 0.11	>50	>50	ND	ND	ND
A-93	13.86 ± 0.19	27.13 ± 1.41	5.89 ± 0.25	15.44 ± 1.98		
A-94	14.05 ± 0.16	>50	<0.08	ND	ND	ND
A-95	14.48 ± 0.15	28.92 ± 0.89	16.33 ± 3.30	ND	ND	ND
A-96	14.70 ± 0.28	30.99 ± 2.10	27.72 ± 2.60	ND	ND	ND
A-97	14.73 ± 0.99	>50	>50	ND	ND	ND
A-98	14.80 ± 0.07	>50	>50	>100	>2	>2
A-99	15.32 ± 0.01	0.98 ± 0.03	1.15 ± 0.25	>100	>102	>86.9
A-100	15.43 ± 0.45	>50	ND	ND	ND	ND
A-101	15.45 ± 0.18	>50	ND	ND	ND	ND
A-102	15.70 ± 0.20	>50	17.98 ± 1.86	ND	ND	ND

A-103	16.41 ± 0.15	4.61 ± 1.65	ND	ND	ND	ND
A-104	16.84 ± 0.04	>50	14.25 ± 0.42	>100		>7.1
A-105	17.44 ± 1.18	33.53 ± 0.57	18.50 ± 2.59	>100	>2.9	>5.4
A-106	17.57 ± 0.65	>50	5.57 ± 1.37	>100	>2	>17.9
A-107	17.66 ± 0.40	1.51 ± 0.06	0.22 ± 0.04	ND	ND	ND
A-108	17.94 ± 0.62	>50	ND	ND	ND	ND
A-109	18.38 ± 0.71	>50	ND	ND	ND	ND
A-110	18.39 ± 0.19	>50	>50	ND	ND	ND
A-111	18.48 ± 0.21	>50	5.24 ± 0.86	81.11 ± 7.49	<1.6	15.5
A-112	19.16 ± 0.79	>50	>50	ND	ND	ND
A-113	19.80 ± 0.23	44.19 ± 3.71	13.30 ± 0.63	ND	ND	ND
A-114	19.80 ± 0.65	0.24 ± 0.00	<0.08	0.40 ± 0.00	1.6	>5
A-115	19.98 ± 0.25	>50	>50	ND	ND	ND
A-116	20.09 ± 0.30	56.10 ± 4.38	11.04 ± 6.18	>100	>1.8	>9
A-117	20.28 ± 0.35	14.65 ± 1.20	32.81 ± 2.29	49.37 ± 8.97	3.4	1.5
A-118	20.56 ± 0.14	54.83 ± 2.01	>50	ND	ND	ND
A-119	21.26 ± 0.71	6.23 ± 0.05	3.55 ± 1.53	13.54 ± 0.90	2.2	3.8
A-120	21.50 ± 0.42	>50	ND	>100	>2	
A-121	22.92 ± 0.85	>50	>50	ND	ND	ND
A-122	23.01 ± 0.35	27.11 ± 0.86	9.55 ± 1.63	ND	ND	ND
A-123	24.08 ± 0.27	>50	ND	ND	ND	ND
A-124	26.78 ± 0.01	>50	49.66 ± 0.88	>100		>2
A-125	28.91 ± 0.03	2.30 ± 0.10	1.13 ± 0.04	2.21 0.05	0.96	1.7
A-126	<10	>50	ND	ND	ND	ND
A-127	5.01 ± 0.46	<2	<10	ND	ND	ND
A-128	<10	>50	ND	ND	ND	ND
